# Supplementary material for: PEG–Lipid–PLGA Hybrid Particles for Targeted Delivery of Anti-Inflammatory Drugs
Source: Pharmaceutics. 2024 Jan 28;16(2):187. doi: 10.3390/pharmaceutics16020187 (PMC10891717; doi:10.3390/pharmaceutics16020187)
Supplement: Supplementary file 1 [file pharmaceutics-16-00187-s001.zip › pharmaceutics-2770169-supplementary.pdf]

Supporting information

PEG–Lipid–PLGA Hybrid Particles for Targeted Delivery of Anti-Inflammatory Drugs

Jana Ismail [1,†], Lea C. Klepsch [1,†], Philipp Dahlke [2], Ekaterina Tsarenko [1], Antje Vollrath [1,3], David Pretzel [1,3], Paul M. Jordan [2,3], Kourosh Rezaei [1], Justyna A. Czaplewska [1,3], Steffi Stumpf [1,3], Baerbel Beringer-Siemers [1,3], Ivo Nischang [1,3,4,5], Stephanie Hoeppener [1,3], Oliver Werz [2,3] and Ulrich S. Schubert [1,3,4,*]

[1] *Laboratory of Organic and Macromolecular Chemistry (IOMC)*
Friedrich Schiller University Jena, Humboldtstraße 10, 07743 Jena, Germany

[2] *Department of Pharmaceutical/Medicinal Chemistry, Institute of Pharmacy*
Friedrich Schiller University Jena, Philosophenweg 14, 07743 Jena, Germany

[3] *Jena Center for Soft Matter (JCSM)*
Friedrich Schiller University Jena, Philosophenweg 7, 07743 Jena, Germany

[4] *Helmholtz Institute for Polymers in Energy Applications Jena (HIPOLE Jena), Lessingstraße 12-14, 07743 Jena, Germany*

[5] *Helmholtz-Zentrum Berlin für Materialien und Energie GmbH (HZB), Hahn-Meitner-Platz 1, 14109 Berlin, Germany*

***** Correspondence: ulrich.schubert@uni-jena.de
† These authors contributed equally to this work.

1 Table of contents

2 Methods 4
2.1 Formulation Method for HNPs 4
2.2 Analyses of Particle Size Distributions in SEM Images Using OpenCV in Python 5
2.3 HPLC Analysis of HNPS 7
2.4 CD14 FITC Staining of Isolated Macrophages 8
3 Materials 9
3.1 HNP components: Polymers, lipids and PEG-Lipids 9
3.2 Cargo molecules (dye and drug) 10
4 Results 11
4.1 HNPs and NLO lLoaded HNPs 11
4.1.1 Formulation 11
4.1.2 Particle characteristics 12
4.1.3 SEC analysis 13
4.1.4 Particle size distribution analysis from SEM images 15
4.1.5 Stability in PBS and in acetate buffer 16

4.1.6 Degradation of HNPs 18
4.1.7 Cytotoxicity studies 19
4.1.8 Uptake studies in M0-MDMs 20
4.2 Dual Loaded PEG-Lipid-PLGA HNPs with Different Sizes and Functionalities 22
4.2.1 Formulation 22
4.2.2 Particle characteristics 23
4.2.3 Particle size distribution analysis from SEM images 26
4.2.4 Free drug analysis *via* SEM measurements 28
4.2.5 Loading capacities of dual loaded HNPs 29
4.2.6 Stability in PBS and in acetate buffer 30
4.2.7 HPLC analysis of dual loaded HNPS and NPs 32
4.2.8 Uptake studies in M1-MDMs 39
4.2.9 Investigation of the inhibition efficacy (5-LOX product formation assay) 40
5 References 40

Index of Figures

Figure S1: Formulation scheme for the preparation of the hybrid nanoparticles (HNP) via single-step nanoprecipitation technique. Image created with BioRender.com, accessed on 2 October 2023. 4
Figure S2: Eluent composition and gradient programming of the developed HPLC method for the analysis of dual-loaded (with the drug BRP-201 and the NLO dye) s-PEG-PLGA NPs and lipid containing s-HNPs. 7
Figure S3: Flow cytometric analysis of CD14 expression of **A**) M_0-MDMs and **B**) M_1-MDMs. Human macrophages were stained with either FITC Mouse IgG2a, κ Isotype Control (Cat. No. 555573; left plot) or FITC Mouse Anti-Human CD14 antibody (Cat. No. 555397/561712/557153; right plot) and analyzed using CytoFlex LX. 8
Figure S4: (**A**) Schematic representation of the structure of the polymer PLGA, lecithin (phosphatidylcholine), and the PEG-Lipid 1,2-distearoyl-sn-glycero-3-phosphoethanolamine (DSPE-PEG_{2000}-X). (**B**) Schematic representation of the structure of different functionalities on the DSPE-PEG. 9
Figure S5: Schematic representation of the chemical structure of (**A**) the dye NLO and (**B**) the drug BRP-201. 10
Figure S6: Stability of HNPs regarding the presence of the surfactant poly(vinyl alcohol) (PVA). 13
Figure S7: Size exclusion chromatography (SEC) analysis of the purified lyophilized HNPs. 13
Figure S8: (**A**) Stability of HNPs and PEG-PLGA NPs in water over four weeks, (**B**) yield after filtration and (**C**) zeta potential in water and 0.01 M sodium chloride (NaCl) solution. 14
Figure S9: Particle size evaluation (number weighted value, d_N) by SEM image processing of dye loaded HNPs after purification. 15
Figure S10: (**A**) Stability of HNPs and PEG-PLGA NPs in PBS buffer over one week and (**B**) stability of HNPs and PEG-PLGA in acetate buffer (Ac.buffer) over one week. 17
Figure S11: Enzymatic degradation of dye loaded HNPs and PEG-PLGA NPs (B-D) as well as dual loaded s- and l-HNPs, as well as s- and l-PEG-PLGA (E-H). HNP-COOH in PBS without proteinase K (A) and

particles mixed with a 1:2 ratio with proteinase K (B to H). Degradation was observed by monitoring the count rate and size by DLS. n = 1. 18
Figure S12: Uptake of the HNPs and PEG-PLGA NPs in M_0-MDMs at three different concentrations (1.8 µg mL^{-1} and 18 µg mL^{-1} with n = 4, 180 µg mL^{-1} with n = 2), reported as MFI and X-fold change as compared to the PEG-PLGA NPs. 20
Figure S13: M_1-MDMs uptake kinetics of the s-HNP-COOH and s-HNP-RGD as compared to s-PEG-PLGA NPs at a concentration of 100 µg mL^{-1} and free NLO in DMSO at 0.06 µg mL^{-1} (representative of the %LC of the HNP) using CLSM over 15 min (scale bar: 10 µm, magnification; 40×). 21
Figure S14: (**A**) Stability of the s-HNPs and s-PEG-PLGA in water over four weeks, (**B**) yield after purification and (**C**) zeta potential in water and 0.01 M sodium chloride (NaCl) solution. 24
Figure S15: (**A**) Stability of l-HNPs and l-PEG-PLGA in water over four weeks, (**B**) yield after purification and (**C**) zeta potential in water and 0.01 M sodium chloride (NaCl) solution. 24
Figure S16: Exact size distribution of s- and l-HNPs, as well as s- and l-PEG-PLGA with the standard deviation. Calculated using$\sigma = \sqrt{PDI} \cdot d_{h,DLS}$.[2] 25
Figure S17: SEM size evaluation of s-HNPs after purification by image processing. 26
Figure S18: SEM size evaluation of l-HNPs by image processing. 27
Figure S19: SEM images of l-HNPs and l-PEG-PLGA NPs before and after filtration through 0.8 µm cellulose acetate filter. White rectangular box indicates the presence of BRP-201 precipitates in the formulations before the filtration procedure. 28
Figure S20: (**A**) BRP-201 and (**B**) NLO loading capacity (LC) of s- and l-HNPs, as well as s- and l-PEG-PLGA NPs. 29
Figure S21: Buffer stability of smaller particles: s-HNPs and s-PEG-PLGA in (**A**) PBS and (**B**) acetate buffer (Ac. buffer). 31
Figure S22: Buffer stability of l-HNPs and l-PEG-PLGA in (**A**) PBS and (**B**) acetate buffer (Ac. buffer). 31
Figure S23: (**A**) Elugram of dual loaded (with the drug BRP-201 and the dye NLO) s-PEG-PLGA NPs recorded by CAD. (**B**) Elugram of s-PEG-PLGA NPs recorded by DAD at 312 nm. The peak at 3.7 min refers to BRP-201. (**C**) Elugram of s-PEG-PLGA NPs recorded by FLD (λ_{ex} = 555 nm, λ_{em} = 592 nm). The peak at 8.4 min refers to NLO. Measurement conditions: Flow rate 0.75 mL min^{-1}, CH_3CN/water with 10 mM ammonium acetate (pH 5.5)/CH_3OH with 10 mM ammonium acetate, gradient conditions can be found in **Figure S2**. 32
Figure S24: (**A**) Elugram of dual loaded (with the drug BRP-201 and the dye NLO) s-HNP-RGD recorded by CAD. (**B**) Elugrams of PLGA, DSPE-PEG-RGD, lecithin, and PVA standards. For simplicity of interpretation, the signal intensities of DSPE-PEG-RGD, lecithin, and PVA are multiplied with a factor of 0.25. (**C**) Elugram of s-HNP-RGD recorded by DAD at 312 nm. Peak at 3.7 min refers to BRP-201. (**D**) Elugram of s-HNP-RGD recorded by FLD (λ_{ex} = 555 nm, λ_{em} = 592 nm). The peak at 8.4 min refers to NLO. Measurement conditions: Flow rate 0.75 mL min^{-1}, CH_3CN/water with 10 mM ammonium acetate (pH 5.5)/CH_3OH with 10 mM ammonium acetate. The gradient conditions can be found in **Figure S2**. 33
Figure S25: (**A**) Elugram of dual loaded (with the drug BRP-201 and the dye NLO) s-HNP-COOH recorded by CAD. (**B**) Elugrams of PLGA, DSPE-PEG-COOH, lecithin, and PVA standards. For simplicity of interpretation, the signal intensities of DSPE-PEG-COOH, lecithin, and PVA are multiplied with a factor of 0.25. (**C**) Elugram of s-HNP-COOH recorded by DAD at 312 nm. Peak at 3.7 min refers to BRP-201. (**D**) Elugram of s-HNP-COOH recorded by FLD (λ_{ex} = 555 nm, λ_{em} = 592 nm). The peak at 8.4 min refers to NLO. Measurement conditions: Flow rate 0.75 mL min^{-1}, CH_3CN/water with 10 mM ammonium acetate (pH 5.5)/ -CH_3OH with 10 mM ammonium acetate. The gradient conditions can be found in **Figure S2**. 34
Figure S26: (**A**) Elugram of dual loaded (with the drug BRP-201 and the dye NLO) s-HNP-COOH/RGD(1:1) recorded by CAD. (**B**) Elugrams of PLGA, DSPE-PEG-COOH, DSPE-PEG-RGD, lecithin,

and PVA standards. For simplicity of interpretation, the signal intensities of DSPE-PEG-COOH, DSPE-PEG-RGD, lecithin, and PVA are multiplied with a factor of 0.25. (**C**) Elugram of s-HNP-COOH/RGD(1:1) recorded by DAD at 312 nm. Peak at 3.7 min refers to BRP-201. (**D**) Elugram of s-HNP-COOH/RGD(1:1) recorded by FLD (λ_{ex} = 555 nm, λ_{em} = 592 nm). The peak at 8.4 min refers to NLO. Measurement conditions: Flow rate 0.75 mL min^{-1}, CH_3CN/water with 10 mM ammonium acetate (pH 5.5)/ -CH_3OH with 10 mM ammonium acetate. The gradient conditions can be found in **Figure S2**. 35
Figure S27: (**A**) Elugram of dual loaded (with the drug BRP-201 and the dye NLO) s-HNP-COOH/RGD(2:1) recorded by CAD. (**B**) Elugrams of PLGA, DSPE-PEG-COOH, DSPE-PEG-RGD, lecithin, and PVA standards. For simplicity of interpretation, the signal intensities of DSPE-PEG-COOH, DSPE-PEG-RGD, lecithin, and PVA are multiplied with a factor of 0.25. (**C**) Elugram of s-HNP-COOH/RGD(2:1) recorded by DAD at 312 nm. Peak at 3.7 min refers to BRP-201. (**D**) Elugram of s-HNP-COOH/RGD(2:1) recorded by FLD (λ_{ex} = 555 nm, λ_{em} = 592 nm). The peak at 8.4 min refers to NLO. Measurement conditions: Flow rate 0.75 mL min^{-1}, CH_3CN/water with 10 mM ammonium acetate (pH 5.5)/CH_3OH with 10 mM ammonium acetate. The gradient conditions can be found in **Figure S2**. 36
Figure S28: (**A**) Calibration curve for BRP-201 and (**B**) double-logarithmic calibration curve for PLGA presented by plotting peak areas as a function of analyte concentrations. Data were fitted linearly. Data were collected at the same elution conditions as shown in **Figure S2**. 37
Figure S29: Elution repeatability experiment by five successive injections of s-HNP-COOH recorded by CAD. Measurement conditions: Flow rate 0.75 mL min^{-1}, CH_3CN/water with 10 mM ammonium acetate (pH 5.5)/CH_3OH with 10 mM ammonium acetate. The gradient conditions can be found in **Figure S2**. 37
Figure S30: Uptake of the s- and l-HNPs as compared to s- and l-PEG-PLGA NPs (P10-P19) in M_1-MDMs at a concentration of 100 µg mL^{-1} (n = 2), reported as X-fold change as compared to the s-PEG-PLGA NPs. 39

2 Methods

2.1 Formulation Method for HNPs

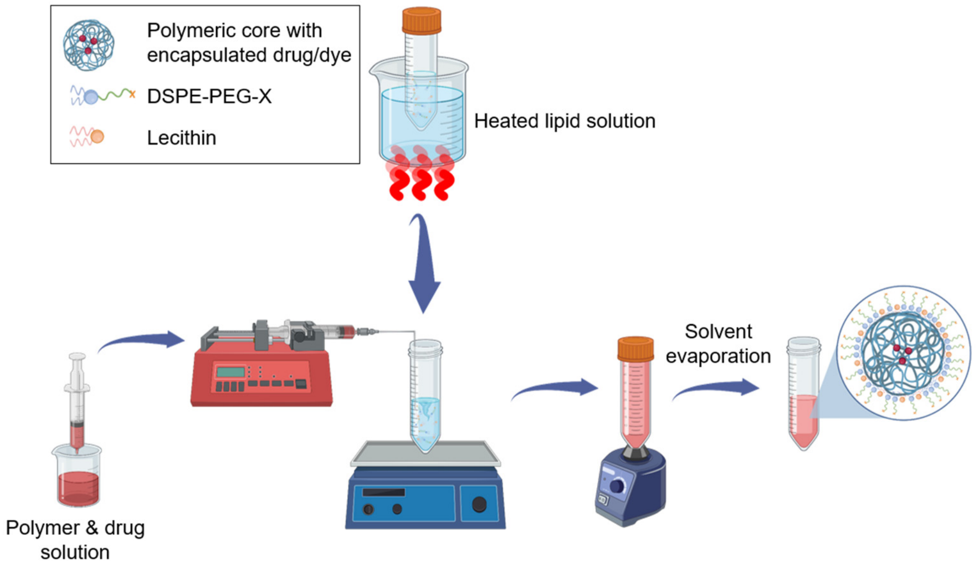

Figure S1: Formulation scheme for the preparation of the hybrid nanoparticles (HNP) via single-step nanoprecipitation technique. Image created with BioRender.com, accessed on 2 October 2023.

2.2 Analyses of Particle Size Distributions in SEM Images Using OpenCV in Python

Analyzing the size distribution of particles in SEM images is a critical task in various scientific and industrial applications. OpenCV in Python offers a powerful set of tools for preprocessing and particle detection. By following the algorithm outlined in this article, the analysis process can be automated leading to the valuable insights about the size distribution of particles within SEM images. Our algorithm involves a series of preprocessing steps and an iterative approach to detect particles until no more are found in the image.

Preprocessing steps

Before we can analyze the particle sizes in SEM images, it is crucial to ensure that the images are properly preprocessed:

1. Histogram equalization: One common preprocessing step is histogram adjustment. It is a method to enhance the contrast in an image. It redistributes the intensity values of pixels in the image to cover the entire intensity range. SEM images can often have varying brightness and contrast, making it difficult to distinguish particles. Histogram adjustment can help improve the image quality.
2. Min-Max normalization: Normalization is a crucial preprocessing step that aims to standardize the intensity values of the SEM image. Min-max scaling transforms pixel values to the range [0, 1]. This step ensures that all images are on a common scale.
3. Adaptive thresholding: After histogram equalization and normalization, we can apply adaptive thresholding to create a binary image. This helps in segmenting the particles from the background. The adaptive thresholding allows us to adaptively determine the threshold for each local region of the image.

Particle Detection Algorithm: Iterative Particle Detection

The heart of the particle size distribution analysis lies in detecting and measuring the particles present in the SEM image. This algorithm works iteratively until no more particles are detected in the image.

Here are the key steps involved:

1. Particle detection: Initial step is to detect particles in the preprocessed image. This can be achieved through contour detection. The contours represent the boundaries of the particles. The better the images are preprocessed in a way that the contrast between particle edges and its surrounding particles or the background are enhanced, the better contour detection step will work which leads to more precise particle detection.

2. Particle measurement: For each detected contour, we can calculate various properties, including the area, perimeter, diameter, and centroid. These properties are crucial for determining the size and location of each particle.

3. Filtering by size: At this point, we can filter out particles based on their size. You can set a size range that is relevant to your analysis. In case of our study we considered the particles with diameters between 10 to 1000 nm. Particles falling within this range are retained, while others are discarded.

4. Masking and removal: After filtering, we create a binary mask that represents the particles we want to retain. This mask is used to remove the detected particles from the image, leaving only the remaining particles for further analysis.

5. Iterative process: Repeat the above steps on the modified image (after removing detected particles) until no more particles are detected. This iterative approach ensures that "all" the particles are accounted for in the analysis.

6. Final size distribution: At the end of the analysis, while having all the desired masks of the particles, we can calculate:

- The diameter of largest possible circle that can be inscribed inside each selected mask
- The diameter of smallest possible circle that can be circumscribed outside each selected mask
- And the average of both above

in pixel number or by having the pixel size of each SEM image in nanometers. By doing this we can be assure of a more robust and trustworthy analysis.

7. Plots: Here, in parallel with displaying the selected contours inside the SEM image as a separate graph, we employed boxplot and histogram to analyze the distribution of the sizes. A boxplot, also known as a box-and-whisker plot, is a graphical representation of the distribution of a dataset. It provides a summary of the key statistical measures, including the median, quartiles, and potential outliers. To construct a boxplot, a rectangular box is drawn, representing the interquartile range (IQR) between the first and third quartiles (Q1 and Q3). A line inside the box denotes the median. Whiskers extend from the box to the minimum and maximum values within a specified range or to a certain multiple of the IQR and the outliers beyond the whiskers. Even though taken into the account for mean values, outliers were individually plotted as circles. Histogram plots illustrates the frequency distribution by dividing the variable range into bins, with bar height representing observation frequency. Both plots provide a comprehensive understanding of the size distribution, aiding a better result interpretation.

2.3 HPLC Analysis of HNPS

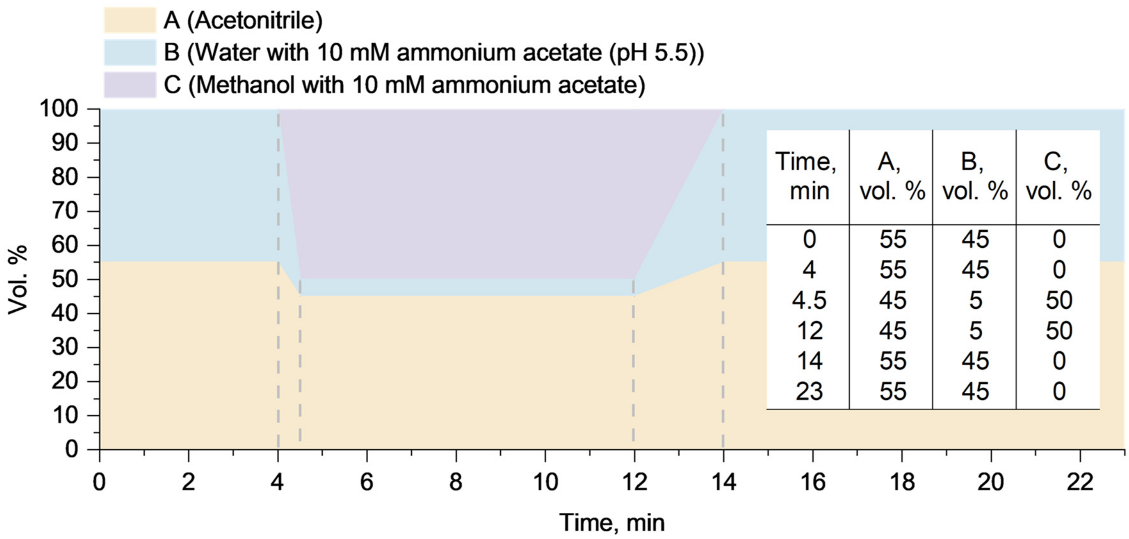

Time, min	A, vol. %	B, vol. %	C, vol. %
0	55	45	0
4	55	45	0
4.5	45	5	50
12	45	5	50
14	55	45	0
23	55	45	0

Figure S2: Eluent composition and gradient programming of the developed HPLC method for the analysis of dual-loaded (with the drug BRP-201 and the NLO dye) s-PEG-PLGA NPs and lipid containing s-HNPs.

2.4 CD14 FITC Staining of Isolated Macrophages

To determine the purity of the isolated macrophages, and clearly discriminate the targeted cells, a cell staining protocol was followed as previously described by Zhang *et al.* (2022). Briefly, prior to staining the cells, non-specific binding of antibodies was blocked by using mouse serum (10 min at 4 °C). Then, cells were stained with either FITC anti-human CD14 (20 µl/test, clone M5E2, catalogue no: 555397, BD Biosciences, Franklin Lakes, NJ, USA) or FITC Mouse IgG2a, κ Isotype Control (Clone G155-178, catalogue no: 554647, BD Biosciences, Heidelberg, Germany) for 20 min at 4 °C.[1] Analysis was performed using the CytoFlex LX (Beckman Coulter GmbH, Krefeld, Germany), and data was analyzed using the CytExpert Software.

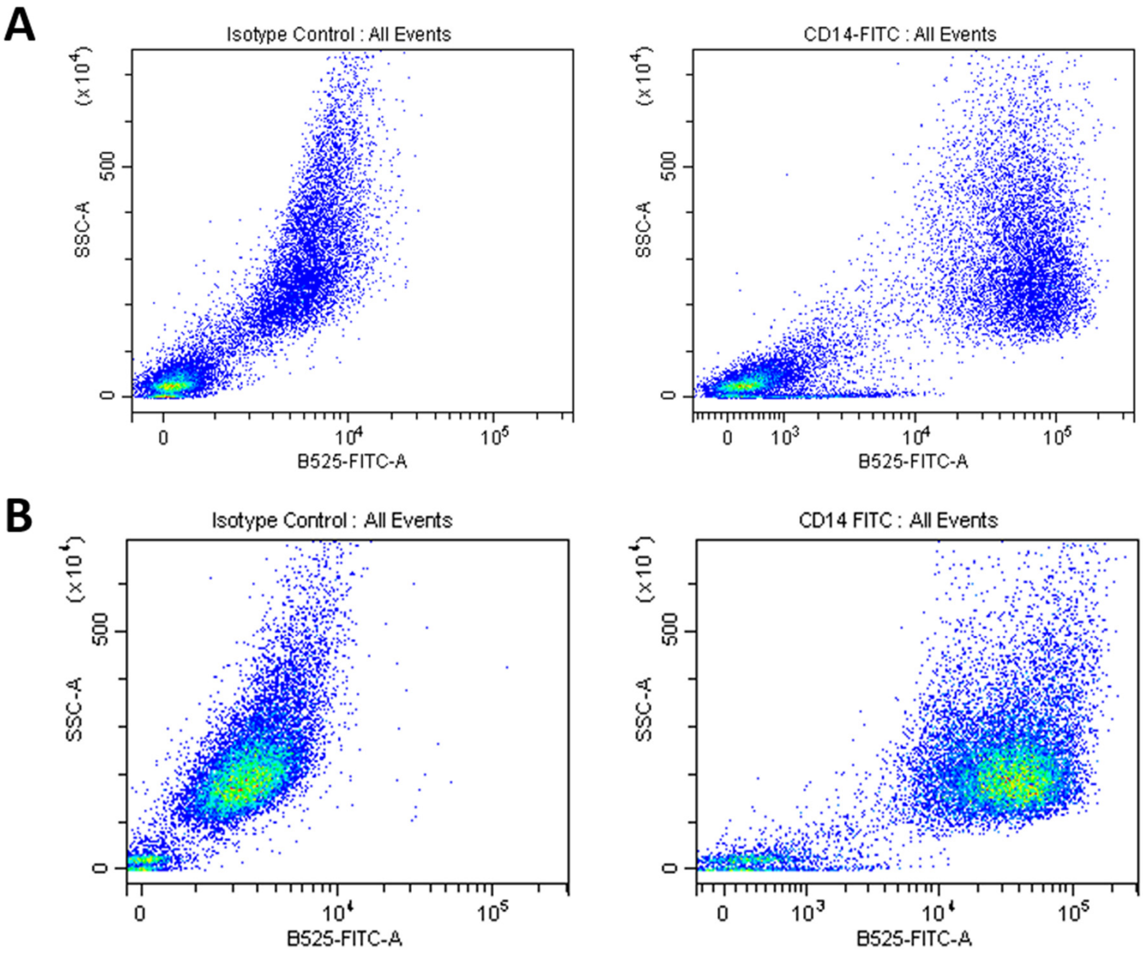

Figure S3: Flow cytometric analysis of CD14 expression of **A**) M_0-MDMs and **B**) M_1-MDMs. Human macrophages were stained with either FITC Mouse IgG2a, κ Isotype Control (Cat. No. 555573; left plot) or FITC Mouse Anti-Human CD14 antibody (Cat. No. 555397/561712/557153; right plot) and analyzed using CytoFlex LX.

3 Materials

3.1 HNP components: Polymers, lipids and PEG-Lipids

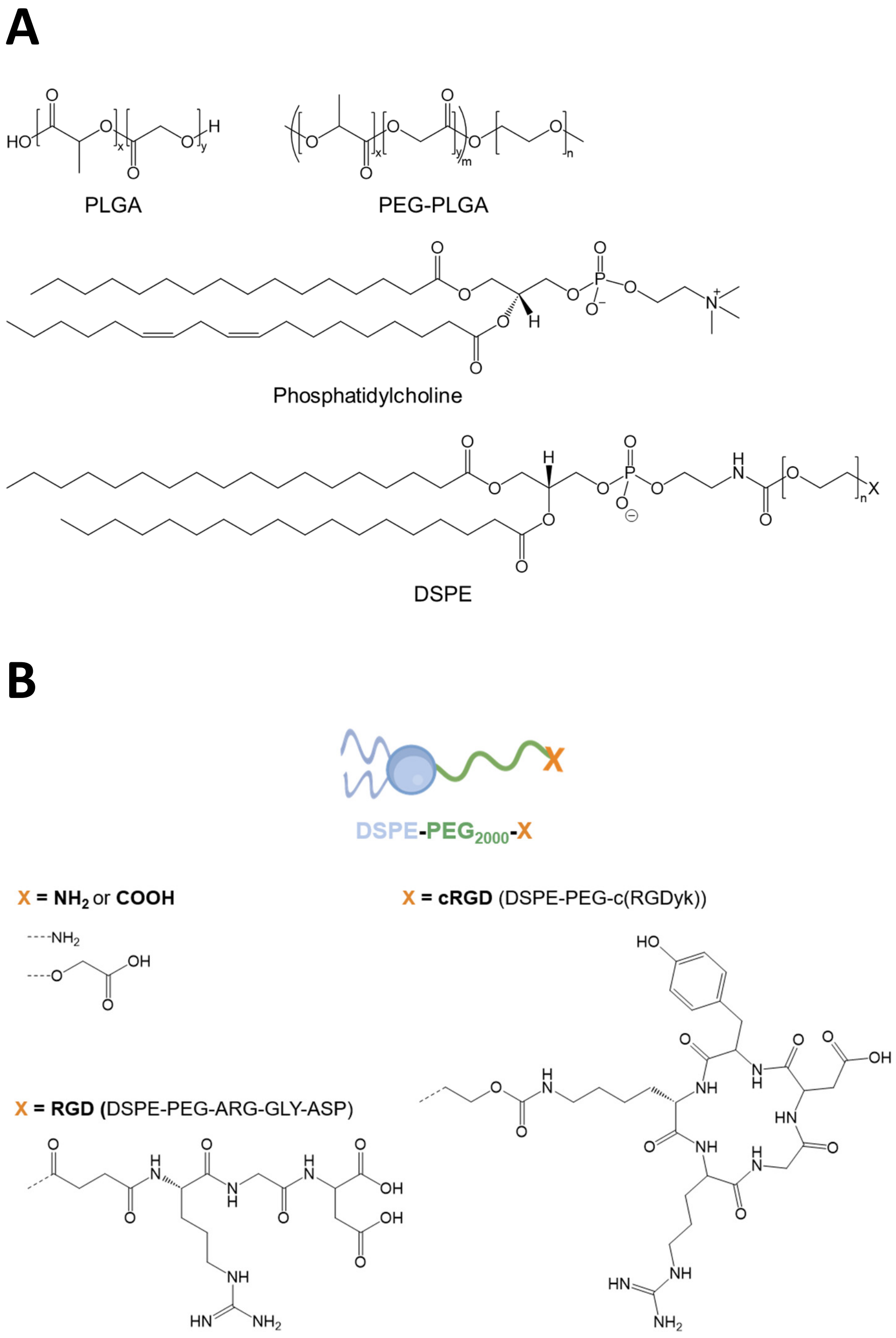

Figure S4: (**A**) Schematic representation of the structure of the polymer PLGA, lecithin (phosphatidylcholine), and the PEG-Lipid 1,2-distearoyl-sn-glycero-3-phosphoethanolamine (DSPE-PEG_{2000}-X). (**B**) Schematic representation of the structure of different functionalities on the DSPE-PEG.

3.2 Cargo molecules (dye and drug)

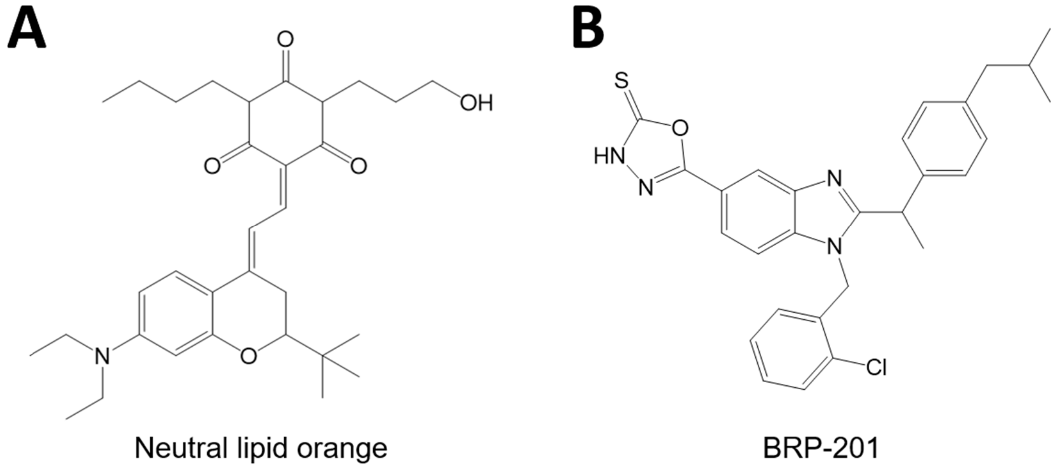

Figure S5: Schematic representation of the chemical structure of (**A**) the dye NLO and (**B**) the drug BRP-201.

4 Results

4.1 HNPs and NLO Loaded HNPs

4.1.1 Formulation

Table S1: Formulation parameters of HNPs and NLO loaded HNPs.

P#	Sample	Polymer	$c_{Polymer}$ [mg mL^{-1}]	PEG-Lipid	Lipid	Cargo	S/W	PVA [wt%]
P1	HNP-NH_2[w/o PVA]	PLGA	2.5	DSPE-PEG-NH_2	Lecithin	-	1:6	-
P2	HNP-COOH[w/o PVA]	PLGA	2.5	DSPE-PEG-COOH	Lecithin	-	1:6	-
P3	HNP-NH_2	PLGA	2.5	DSPE-PEG-NH_2	Lecithin	-	1:6	5
P4	HNP-COOH	PLGA	2.5	DSPE-PEG-COOH	Lecithin	-	1:6	5
**NLO loaded HNPs**								
[a]P5	HNP-NH_2	PLGA	2.5	DSPE-PEG-NH_2	Lecithin	NLO	1:6	10
[a]P6	HNP-COOH	PLGA	2.5	DSPE-PEG-COOH	Lecithin	NLO	1:6	5
[a]P7	HNP-RGD	PLGA	2.5	DSPE-PEG-RGD	Lecithin	NLO	1:6	5
[a]P8	HNP-cRGD	PLGA	2.5	DSPE-PEG-cRGD	Lecithin	NLO	1:6	5
[a]P9	PEG-PLGA	PEG-PLGA:PLGA(1:2)	2.5	-	-	NLO	1:6	5

Solvent to water ratio (S/W). The polymer was dissolved in CH_3CN, the dye was dissolved in DMSO, the lipids were dissolved in a 4 wt% ethanol in water solution. The lipid to polymer ratio (L/P ratio) was always 15 wt% referred to the polymer mass. The initial amount of dye NLO was 0.1 wt%. [a]Formulation performed with n = 5.

4.1.2 Particle characteristics

Table S2: DLS and ELS data, stability over time in water, yield and LC values from HNPs and NLO loaded HNPs.

P#	Sample	d_H [nm] (PDI) after purification	ζ in water [mV]	ζ in NaCl [mV]	d_H [nm] (PDI) after 2 weeks	d_H [nm] (PDI) after 4 weeks	Yield [%]	LC_{NLO} [%]	d_H [nm] (PDI) after filtration $^{n=1}$	LC_{NLO} after filtration [%] $^{n=1}$
P1	HNP-NH_2	138 (0.26)	−25	−2	146 (0.26)	403 (0.38)	44	-	-	-
P2	HNP-COOH	130 (0.18)	−38	−22	136 (0.14)	129 (0.16)	58	-	-	-
P3	HNP-NH_2[w/o PVA]	153 (0.28)	−11	−2	189 (0.32)	802 (0.58)	27	-	-	-
P4	HNP-COOH[w/o PVA]	126 (0.17)	−40	−34	127 (0.16)	124 (0.15)	71	-	-	-
**NLO loaded HNPs**										
[a]P5	HNP-NH_2	157(0.17)	−20	−2	152 (0.17)	166 (0.17)	47	0.06	143 (0.09)	0.06
[a]P6	HNP-COOH	146 (0.13)	−35	−19	140 (0.12)	139 (0.13)	67	0.08	133 (0.15)	0.07
[a]P7	HNP-RGD	146 (0.14)	−35	−14	140 (0.13)	140 (0.13)	72	0.08	131 (0.11)	0.06
[a]P8	HNP-cRGD	166 (0.24)	−29	−6	151 (0.18)	147 (0.16)	41	0.07	141 (0.16)	0.07
[a]P9	PEG-PLGA	118 (0.08)	−14	−4	116 (0.08)	115 (0.07)	64	0.05	116 (0.07)	0.05

Hydrodynamic diameter (d_H), polydispersity index (PDI), zeta potential (ζ), loading capacity (LC). [a]Formulation performed with n = 5.

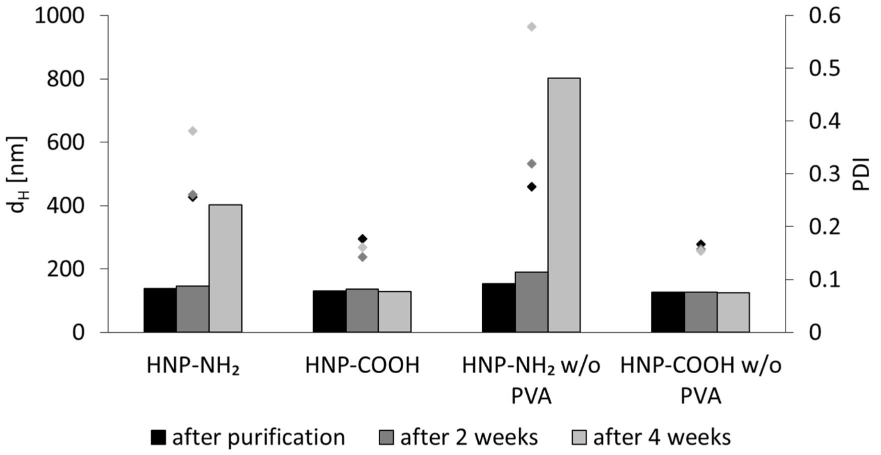

Figure S6: Stability of HNPs regarding the presence of the surfactant poly(vinyl alcohol) (PVA).

4.1.3 SEC analysis

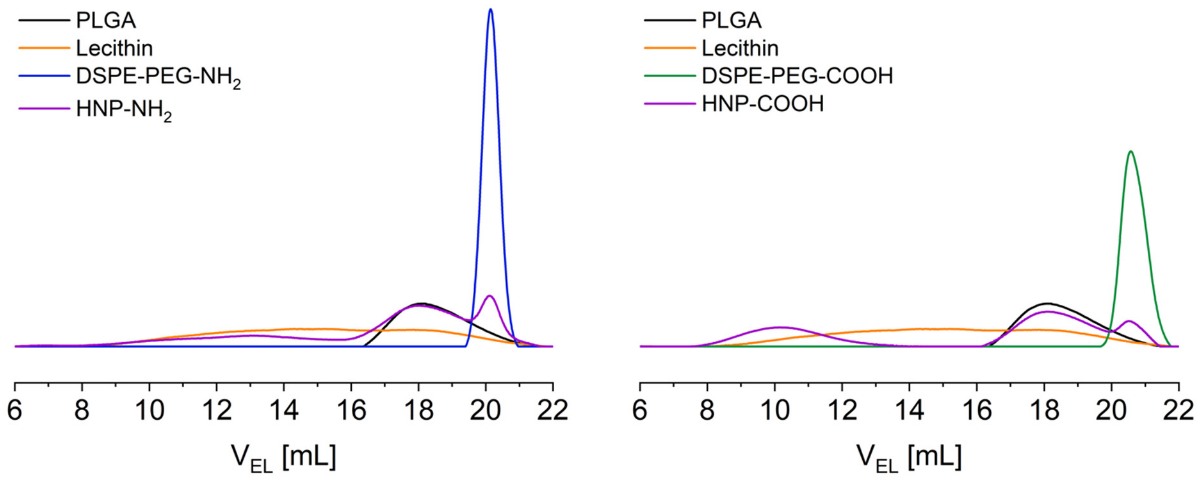

Figure S7: Size exclusion chromatography (SEC) analysis of the purified lyophilized HNPs.

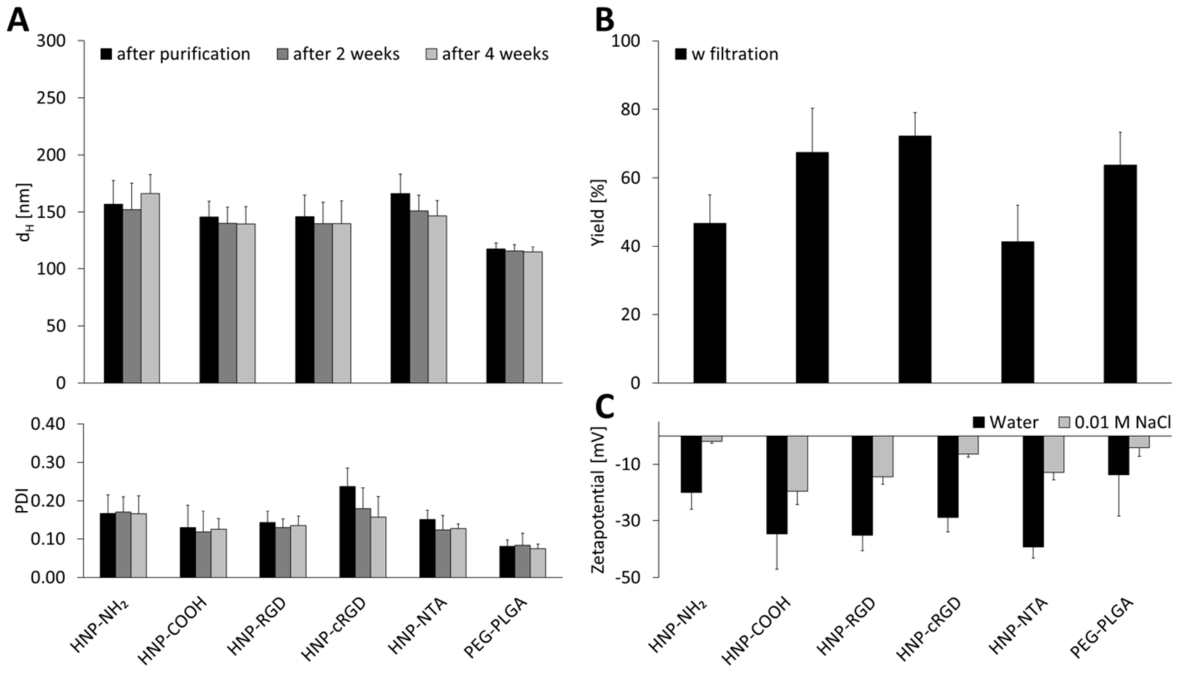

Figure S8: (**A**) Stability of HNPs and PEG-PLGA NPs in water over four weeks, (**B**) yield after filtration and (**C**) zeta potential in water and 0.01 M sodium chloride (NaCl) solution.

4.1.4 Particle size distribution analysis from SEM images

HNP-NH_2

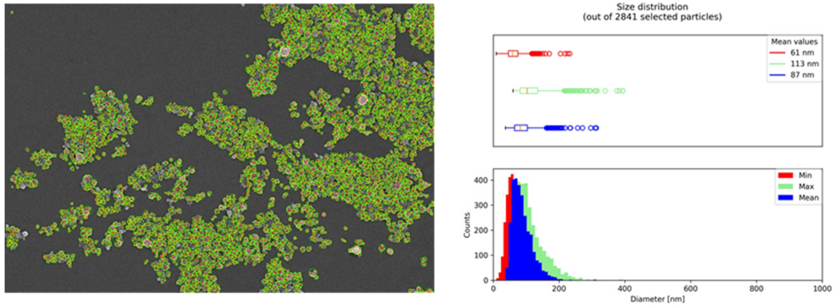

HNP-COOH

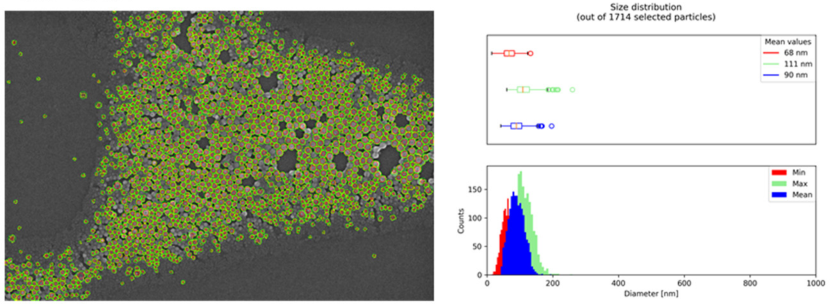

HNP-RGD

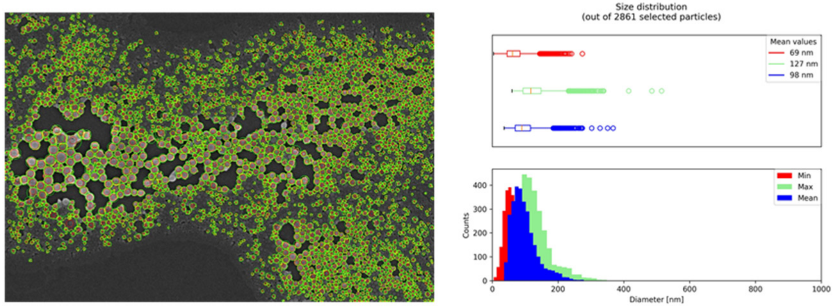

HNP-cRGD

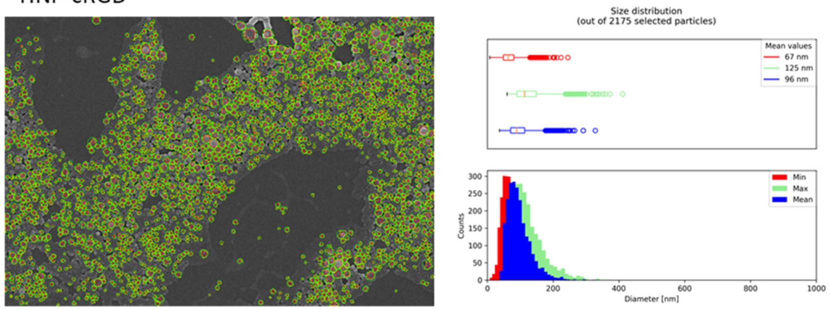

PEG-PLGA

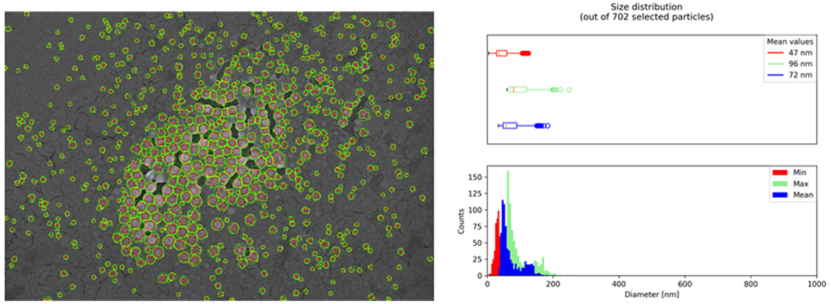

Figure S9: Particle size evaluation (number weighted value, d_N) by SEM image processing of dye loaded HNPs after purification.

4.1.5 Stability in PBS and in acetate buffer

Table S3: DLS data of all formulations measured in PBS and in acetate buffer (Ac. buffer).

P#	Sample	d_H [nm] (PDI) PBS	d_H [nm] (PDI) PBS 24 h	d_H [nm] (PDI) PBS 1 week	d_H [nm] (PDI) Ac.buffer	d_H [nm] (PDI) Ac.buffer 24 h	d_H [nm] (PDI) Ac.buffer 1 week
P1	HNP-NH_2[w/o PVA]	154 (0.25)	-	-	130 (0.20)	-	-
P2	HNP-COOH[w/o PVA]	126 (0.12)	-	-	128 (0.10)	-	-
P3	HNP-NH_2	1136 (0.14)	-	-	138 (0.22)	-	-
P4	HNP-COOH	119 (0.11)	-	-	124 (0.10)	-	-
**NLO loaded HNPs**							
[a]P5	HNP-NH_2	150 (0.09)	203 (0.31)	225 (0.38)	141 (0.09)	153 (0.07)	157 (0.10)
[a]P6	HNP-COOH	130 (0.09)	130 (0.09)	126 (0.08)	134 (0.08)	131 (0.07)	128 (0.08)
[a]P7	HNP-RGD	139 (0.11)	135 (0.06)	129 (0.07)	242 (0.09)	139 (0.08)	138 (0.10)
[a]P8	HNP-cRGD	146 (0.15)	139 (0.17)	139 (0.15)	161 (0.17)	183 (0.18)	197 (0.21)
[a]P9	PEG-PLGA	116 (0.05)	119 (0.07)	118 (0.08)	116 (0.05)	119 (0.06)	120 (0.07)

Hydrodynamic diameter (d_H), polydispersity index (PDI), phosphate-buffered saline (PBS), acetate buffer (Ac.buffer). [a]Formulation performed with n = 5.

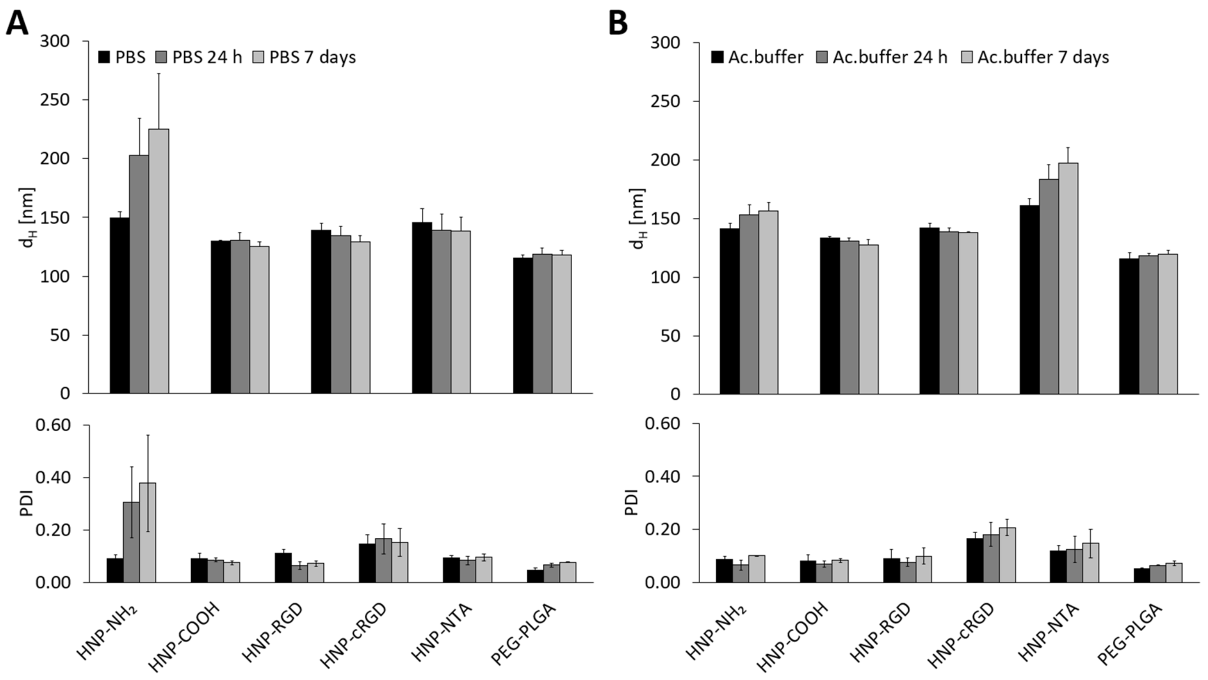

Figure S10: (**A**) Stability of HNPs and PEG-PLGA NPs in PBS buffer over one week and (**B**) stability of HNPs and PEG-PLGA in acetate buffer (Ac.buffer) over one week.

4.1.6 Degradation of HNPs

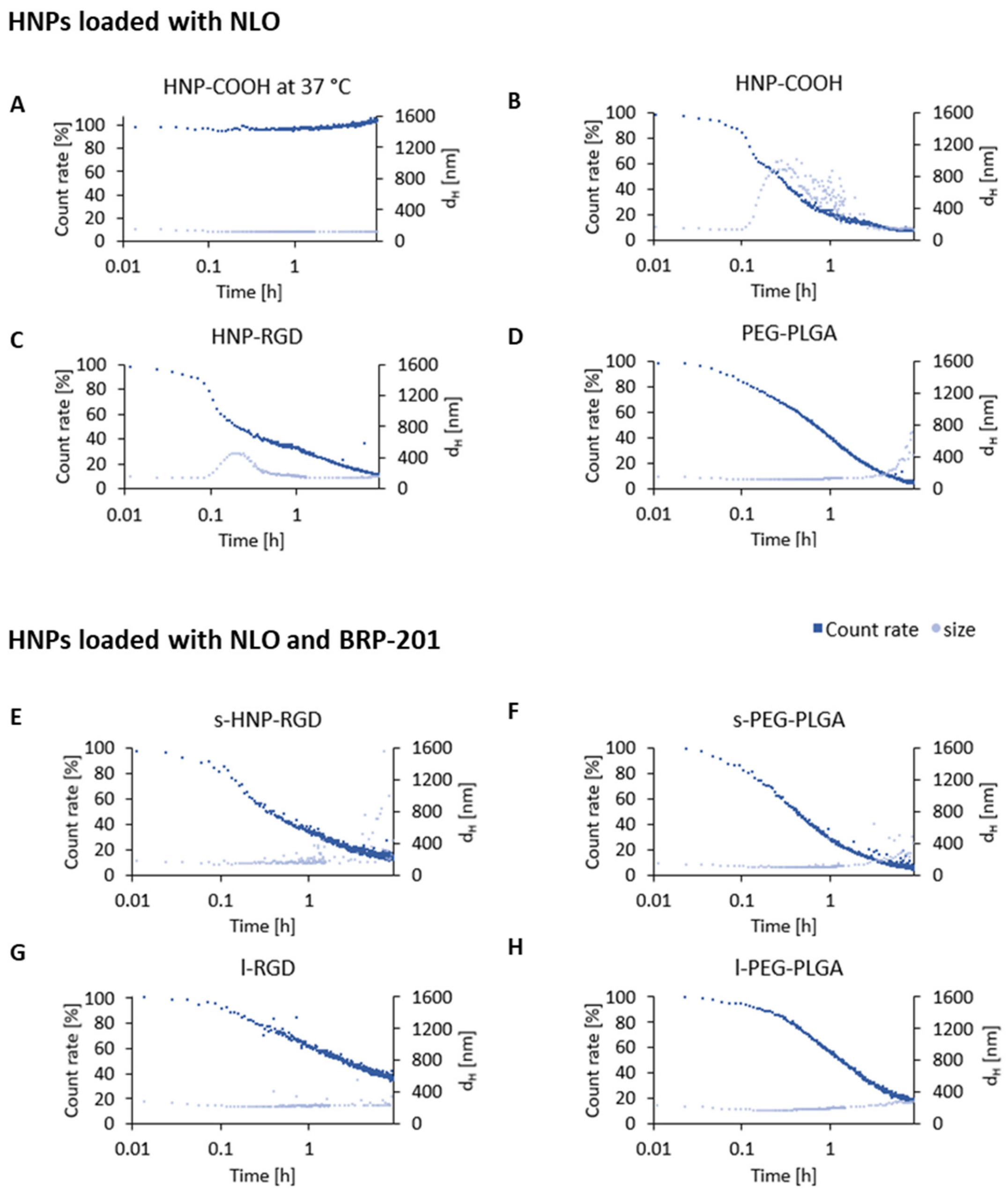

Figure S11: Enzymatic degradation of dye loaded HNPs and PEG-PLGA NPs (B-D) as well as dual loaded s- and l-HNPs, as well as s- and l-PEG-PLGA (E-H). HNP-COOH in PBS without proteinase K (A) and particles mixed with a 1:2 ratio with proteinase K (B to H). Degradation was observed by monitoring the count rate and size by DLS. n = 1.

4.1.7 Cytotoxicity studies

Table S4: Measurement data of the cytotoxicity studies of dye loaded HNPs and PEG-PLGA nanoparticles on M_0-MDMs at three different concentrations (180, 18 and 1.8 µg mL^{-1}) after 24 h reported as average metabolic activity [%]. n = 4 of one formulation batch.

P#	Sample	Metabolic Activity [%] c_{HNP} = 1.8 µg mL^{-1}	Metabolic Activity [%] c_{HNP} = 18 µg mL^{-1}	Metabolic Activity [%] c_{HNP} = 180 µg mL^{-1}
P5	HNP-NH_2	104.0	94.4	103.9
P6	HNP-COOH	100.3	98.0	102.7
P7	HNP-RGD	98.9	94.0	98.2
P8	HNP-cRGD	97.2	99.3	104.3
P9	PEG-PLGA	102.0	101.7	104.2

4.1.8 Uptake studies in M0-MDMs

Table S5: Measurement data of the uptake studies of dye loaded HNPs and PEG-PLGA NPs in M_0-MDMs at three different concentrations (1.8 and 18 µg mL^{-1} with n = 4, 180 µg mL^{-1} with n = 2) reported as mean fluorescence intensity (MFI) corrected for the fluorescence of the particle samples.

P#	Sample	Corrected MFI c_{HNP} = 1.8 µg mL^{-1}	Corrected MFI c_{HNP} = 18 µg mL^{-1}	Corrected MFI c_{HNP} = 180 µg mL^{-1}
P5	HNP-NH_2	43750	394924	3759375
P6	HNP-COOH	39956	300502	3971806
P7	HNP-RGD	39264	234807	3288117
P8	HNP-cRGD	42565	290119	2772436
P9	PEG-PLGA	2474	138081	1285226

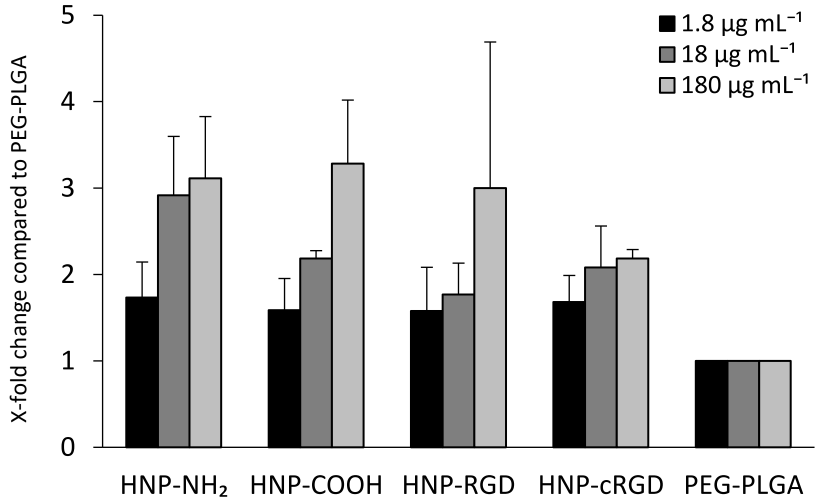

Figure S12: Uptake of the HNPs and PEG-PLGA NPs in M_0-MDMs at three different concentrations (1.8 µg mL^{-1} and 18 µg mL^{-1} with n = 4, 180 µg mL^{-1} with n = 2), reported as MFI and X-fold change as compared to the PEG-PLGA NPs.

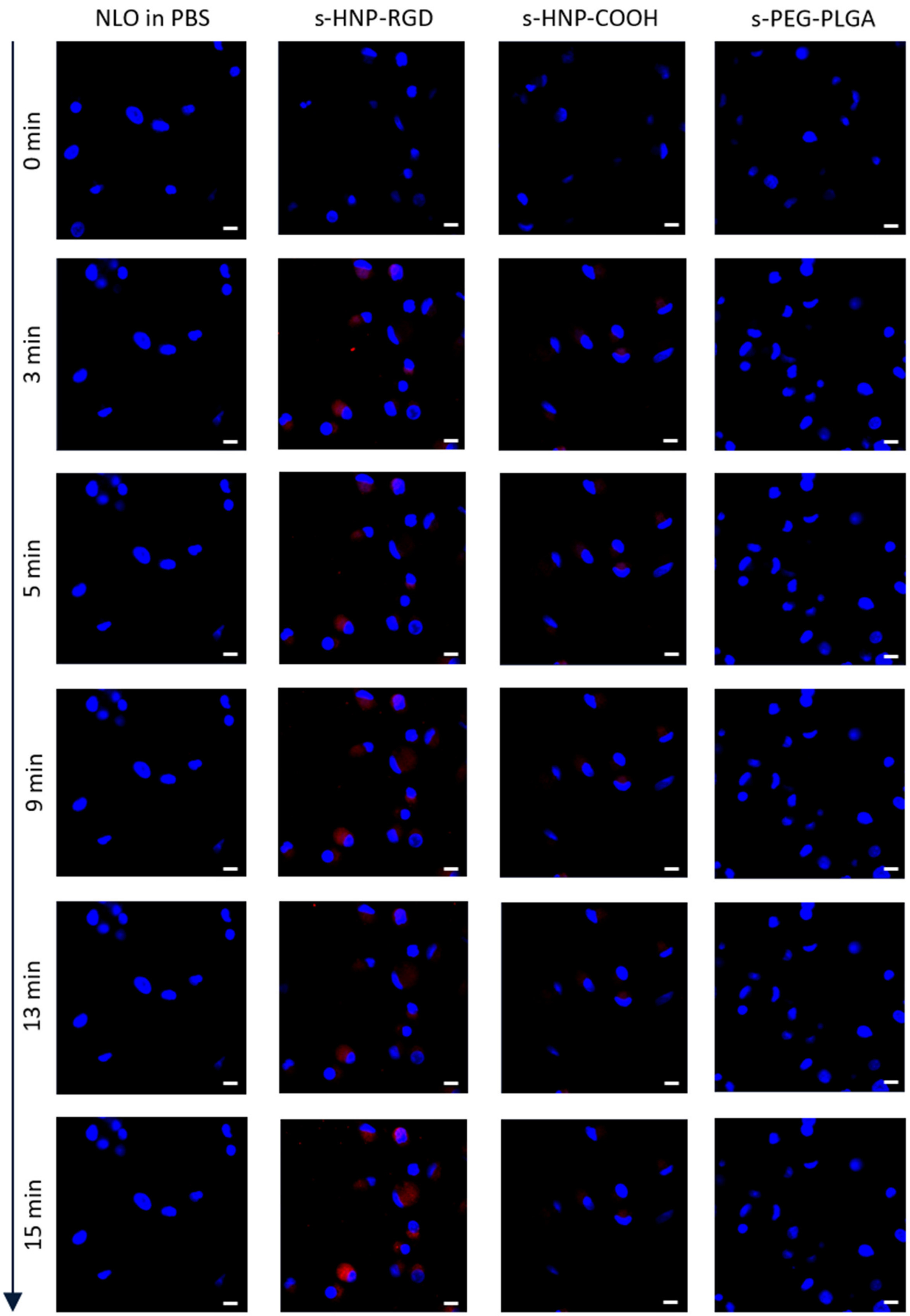

Figure S13: M_1-MDMs uptake kinetics of the s-HNP-COOH and s-HNP-RGD as compared to s-PEG-PLGA NPs at a concentration of 100 µg mL^{-1} and free NLO in DMSO at 0.06 µg mL^{-1} (representative of the %LC of the HNP) using CLSM over 15 min (scale bar: 10 µm, magnification; 40×).

4.2 Dual Loaded PEG-Lipid-PLGA HNPs with Different Sizes and Functionalities

4.2.1 Formulation

Table S6: Formulation parameters of HNPs loaded with BRP-201 and NLO dye.

P#	Sample	Polymer	$c_{Polymer}$ [mg mL^{-1}]	PEG-Lipid	Lipid	Cargo	S/W	PVA [wt%]
**Smaller particles**								
[a]P10	s-HNP-COOH	PLGA	2.5	DSPE-PEG-COOH	Lecithin	BRP-201 + NLO	1:6	25
[a]P11	s-HNP-RGD	PLGA	2.5	DSPE-PEG-RGD	Lecithin	BRP-201 + NLO	1:6	25
[a]P12	s-HNP-COOH/RGD(1:1)	PLGA	2.5	DSPE-PEG-COOH: DSPE-PEG-RGD (1:1)	Lecithin	BRP-201 + NLO	1:6	25
[a]P13	s-HNP-COOH/RGD(2:1)	PLGA	2.5	DSPE-PEG-COOH: DSPE-PEG-RGD (2:1)	Lecithin	BRP-201 + NLO	1:6	25
[a]P14	s-PEG-PLGA	PEG-PLGA:PLGA(1:2)	2.5	-	-	BRP-201 + NLO	1:6	25
**Larger particles**								
[a]P15	l-HNP-COOH	PLGA	25	DSPE-PEG-COOH	Lecithin	BRP-201 + NLO	1:7.5	25
[a]P16	l-HNP-RGD	PLGA	25	DSPE-PEG-RGD	Lecithin	BRP-201 + NLO	1:7.5	25
[a]P17	l-HNP-COOH/RGD(1:1)	PLGA	25	DSPE-PEG-COOH: DSPE-PEG-RGD (1:1)	Lecithin	BRP-201 + NLO	1:7.5	25
[a]P18	HNP-COOH/RGD(2:1)	PLGA	25	DSPE-PEG-COOH: DSPE-PEG-RGD (2:1)	Lecithin	BRP-201 + NLO	1:7.5	25
[a]P19	l-PEG-PLGA	PEG-PLGA:PLGA(1:2)	25	-	-	BRP-201 + NLO	1:7.5	25

Solvent-to-water ratio (S/W). The polymer was dissolved in CH_3CN, the drug and dye were dissolved in DMSO, the lipids were dissolved in a 4 wt% ethanol in water solution. The lipid to polymer ratio (L/P ratio) was always 15 wt% referred to the polymer mass. The initial amount of dye NLO was 0.1 wt% and the initial amount of the drug BRP-201 was 3 wt%. [a]Formulation performed with n = 3.

4.2.2 Particle characteristics

Table S7: DLS and ELS data, stability over time in water, yield and LC values from all HNPs loaded with BRP-201 and NLO.

P#	Sample	d_H [nm] (PDI) after purification	ζ in water [mV]	ζ in NaCl [mV]	d_H [nm] (PDI) after 2 weeks	d_H [nm] (PDI) after 4 weeks	Yield [%]	LC_{NLO} [%]	$LC_{BRP\text{-}201}$ [%]	d_H [nm] (PDI) after filtration	LC_{NLO} after filtration [%]	$LC_{BRP\text{-}201}$ after filtration [%]
**Smaller particles**												
[a]P10	s-HNP-COOH	137 (0.23)	−24	−0.3	118 (0.19)	115 (0.20)	60	0.06	1.33	111 (0.05)	0.03	0.40
[a]P11	s-HNP-RGD	153 (0.32)	−40	−11	171 (0.29)	157 (0.27)	52	0.07	1.24	141 (0.08)	0.06	0.40
[a]P12	s-HNP-COOH/RGD(1:1)	143 (0.27)	−36	−8	152 (0.27)	150 (0.23)	62	0.06	1.00	138 (0.06)	0.05	0.38
[a]P13	s-HNP-COOH/RGD(2:1)	139 (0.24)	−41	−6	150 (0.23)	144 (0.20)	52	0.07	1.07	138 (0.08)	0.05	0.47
[a]P14	s-PEG-PLGA	142 (0.25)	−35	−5	151 (0.26)	148 (0.23)	53	0.07	1.15	138 (0.11)	0.05	0.43
**Larger particles**												
[a]P15	l-HNP-COOH	174 (0.21)	−24	−1	178 (0.14)	176 (0.13)	51	0.11	2.89	173 (0.08)	0.08	0.99
[a]P16	l-HNP-RGD	249 (0.29)	−41	−8	249 (0.17)	247 (0.17)	46	0.08	1.37	221 (0.07)	0.08	0.49
[a]P17	l-HNP-COOH/RGD(1:1)	234 (0.25)	−34	−7	240 (0.18)	238 (0.16)	53	0.08	1.38	222 (0.09)	0.09	0.62
[a]P18	l-HNP-COOH/RGD(2:1)	256 (0.28)	−35	−6	256 (0.21)	250 (0.18)	51	0.08	1.70	222 (0.05)	0.08	0.70
[a]P19	l-PEG-PLGA	252 (0.27)	−35	−4	249 (0.17)	247 (0.19)	56	0.07	1.38	222 (0.10)	0.08	0.94

Hydrodynamic diameter (d_H), polydispersity index (PDI), zeta potential (ζ), loading capacity (LC). [a]Formulation performed with n = 3.

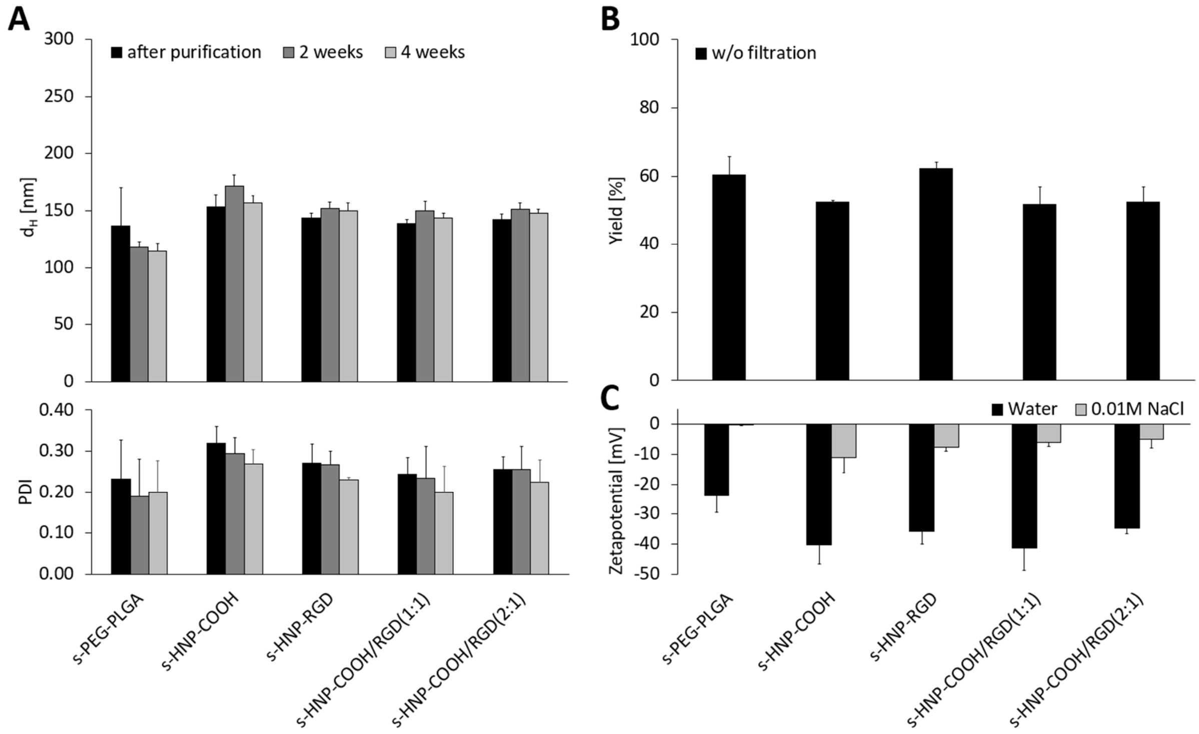

Figure S14: (**A**) Stability of the s-HNPs and s-PEG-PLGA in water over four weeks, (**B**) yield after purification and (**C**) zeta potential in water and 0.01 M sodium chloride (NaCl) solution.

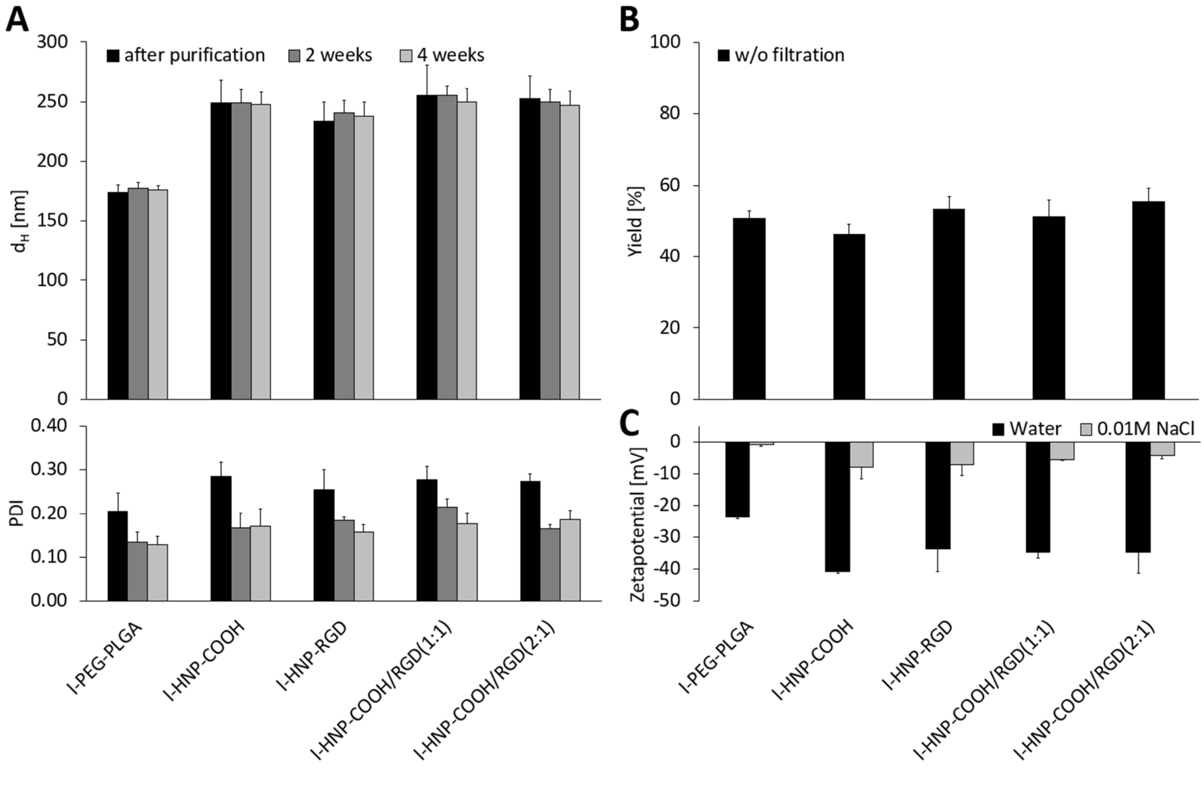

Figure S15: (**A**) Stability of l-HNPs and l-PEG-PLGA in water over four weeks, (**B**) yield after purification and (**C**) zeta potential in water and 0.01 M sodium chloride (NaCl) solution.

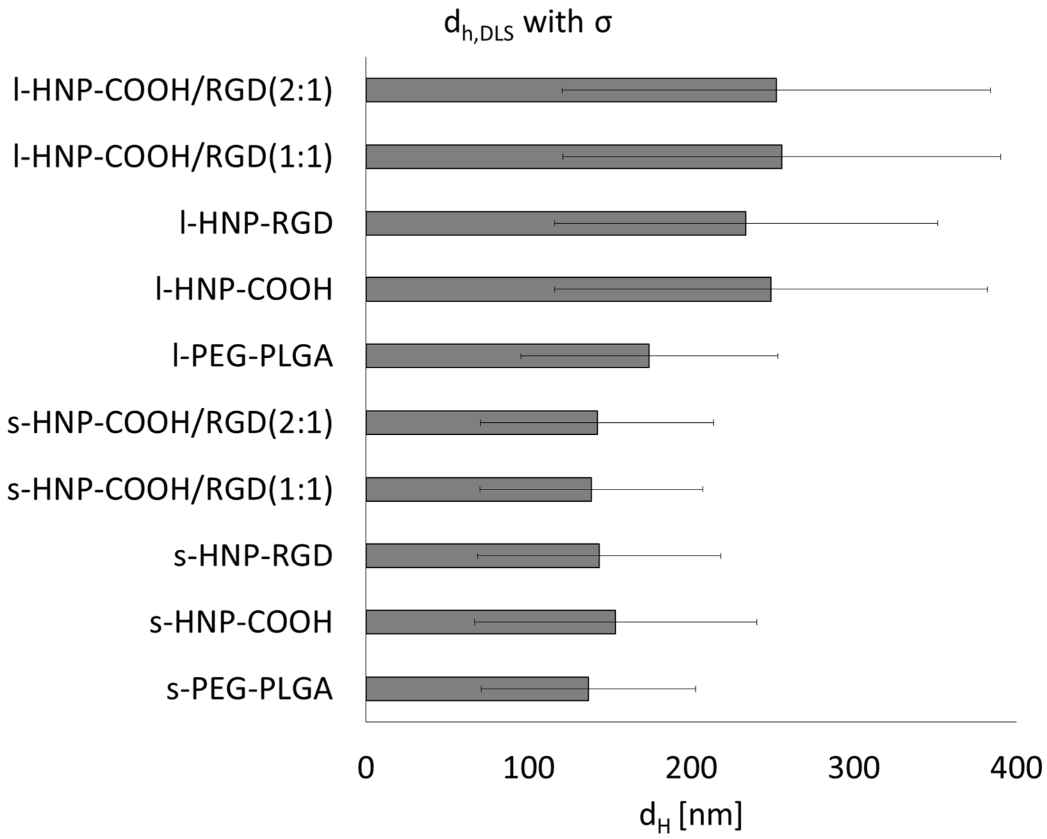

Figure S16: Exact size distribution of s- and l-HNPs, as well as s- and l-PEG-PLGA with the standard deviation. Calculated using $\sigma = \sqrt{PDI} \cdot d_{h,DLS}$.[2]

4.2.3 Particle size distribution analysis from SEM images

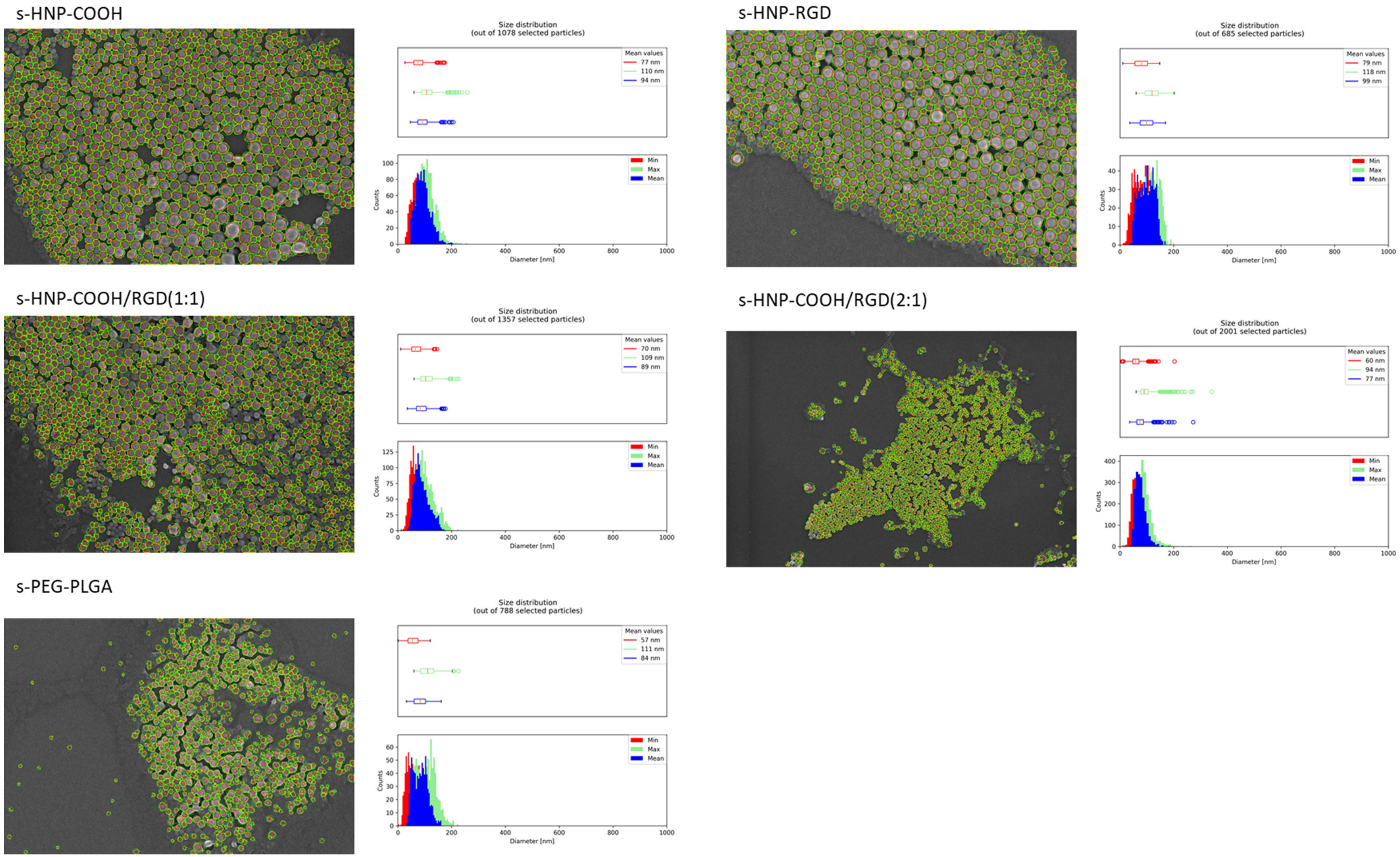

Figure S17: SEM size evaluation of s-HNPs after purification by image processing.

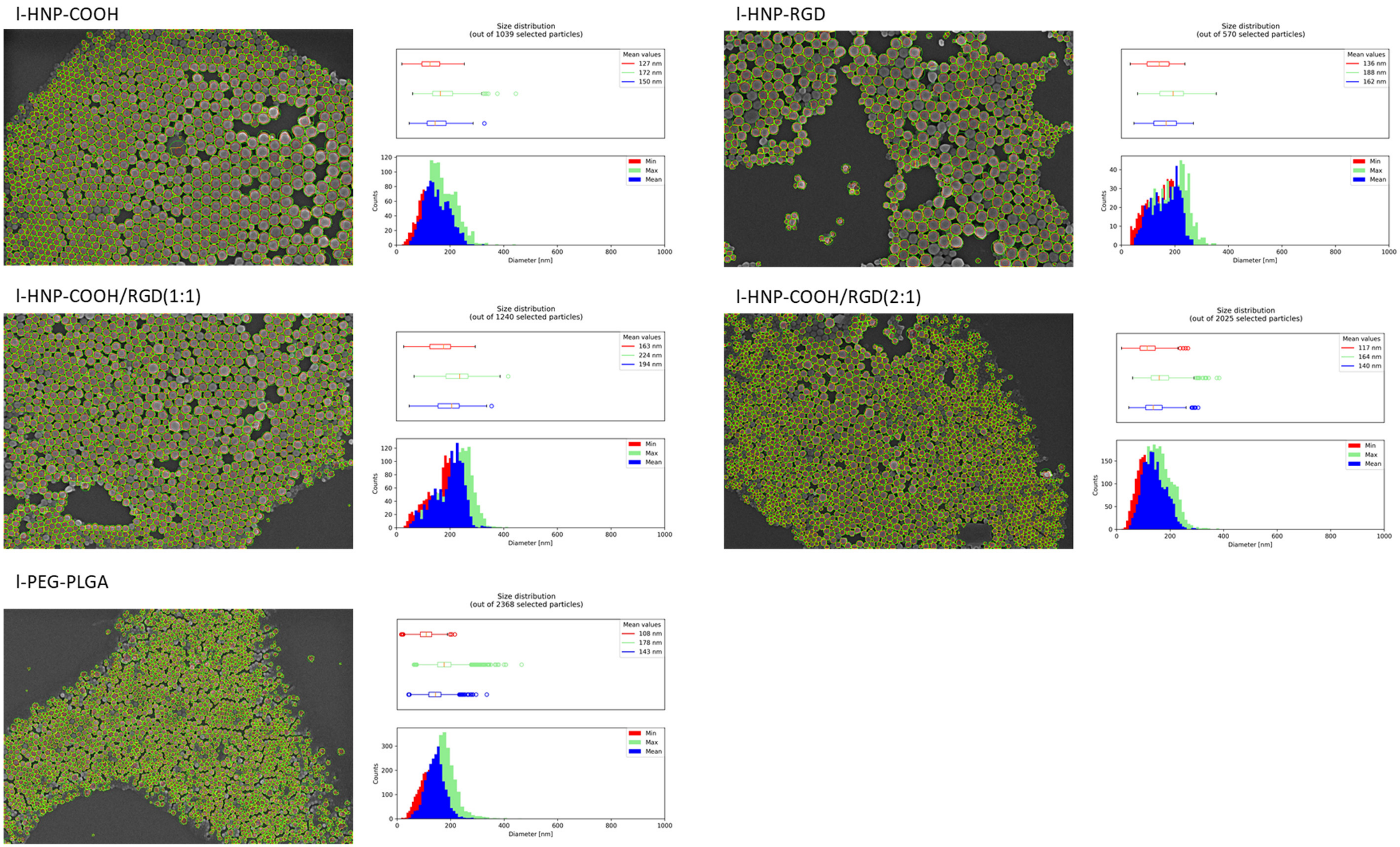

Figure S18: SEM size evaluation of l-HNPs by image processing.

4.2.4 Free drug analysis *via* SEM measurements

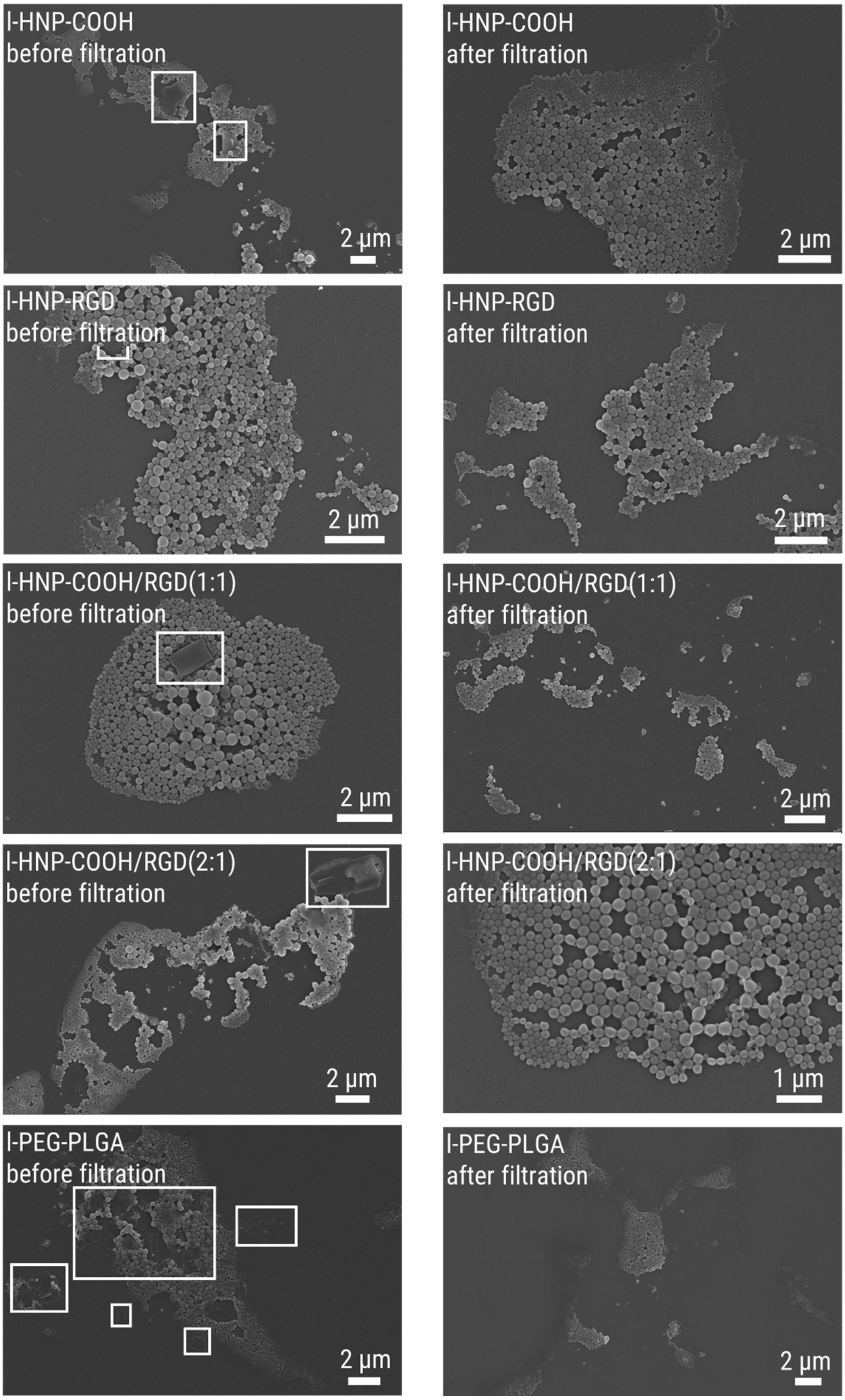

Figure S19: SEM images of l-HNPs and l-PEG-PLGA NPs before and after filtration through 0.8 µm cellulose acetate filter. White rectangular box indicates the presence of BRP-201 precipitates in the formulations before the filtration procedure.

4.2.5 Loading capacities of dual loaded HNPs

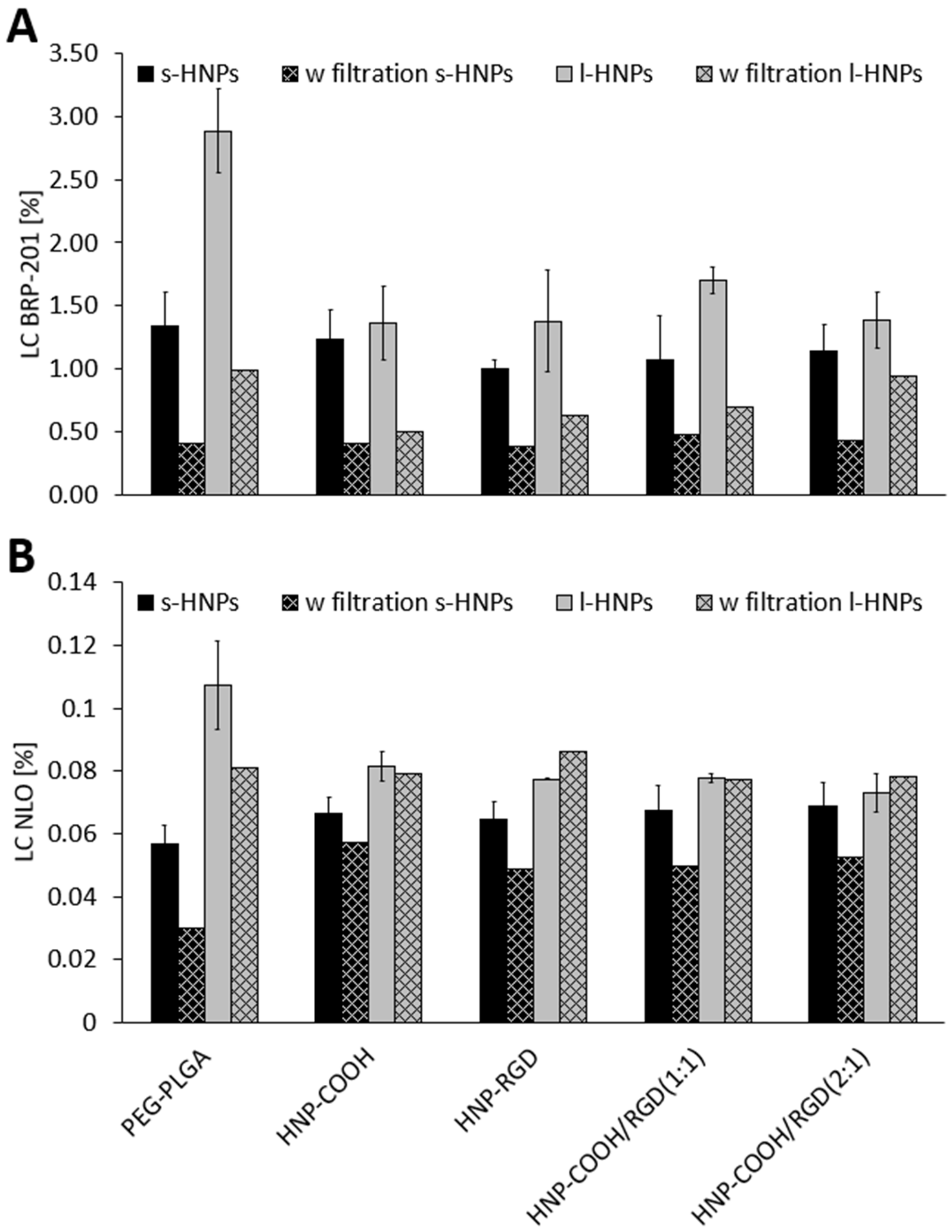

Figure S20: (**A**) BRP-201 and (**B**) NLO loading capacity (LC) of s- and l-HNPs, as well as s- and l-PEG-PLGA NPs.

4.2.6 Stability in PBS and in acetate buffer

Table S8: DLS data of HNP formulations measured in PBS and in acetate buffer (Ac. buffer).

P#	Sample	d_H [nm] (PDI) PBS 48 h	d_H [nm] (PDI) PBS 1 weeks	d_H [nm] (PDI) PBS 3 weeks	d_H [nm] (PDI) Ac.buffer 48 h	d_H [nm] (PDI) Ac.buffer 1 weeks	d_H [nm] (PDI) Ac.buffer 3 weeks
**Smaller particles**							
[a]P10	s-HNP-COOH	121 (0.19)	115 (0.14)	116 (0.13)	121 (0.16)	120 (0.15)	117 (0.12)
[a]P11	s-HNP-RGD	157 (0.27)	158 (0.26)	155 (0.26)	158 (0.27)	156 (0.26)	155 (0.27)
[a]P12	s-HNP-COOH/RGD(1:1)	143 (0.24)	143 (0.19)	146 (0.20)	148 (0.23)	146 (0.19)	146 (0.21)
[a]P13	s-HNP-COOH/RGD(2:1)	144 (0.22)	142 (0.20)	143 (0.19)	145 (0.21)	143 (0.18)	151 (0.20)
[a]P14	s-PEG-PLGA	144 (0.23)	143 (0.21)	145 (0.22)	145 (0.24)	145 (0.22)	147 (0.20)
**Larger particles**							
[a]P15	l-HNP-COOH	172 (0.11)	166 (0.10)	172 (0.10)	171 (0.12)	169 (0.10)	169 (0.13)
[a]P16	l-HNP-RGD	227 (0.18)	225 (0.14)	240 (0.13)	235 (0.16)	234 (0.15)	238 (0.15)
[a]P17	l-HNP-COOH/RGD(1:1)	227 (0.16)	225 (0.13)	231 (0.13)	232 (0.14)	232 (0.14)	234 (0.16)
[a]P18	l-HNP-COOH/RGD(2:1)	239 (0.15)	239 (0.18)	248 (0.16)	246 (0.17)	249 (0.18)	251 (0.16)
[a]P19	l-PEG-PLGA	232 (0.20)	234 (0.15)	235 (0.15)	239 (0.17)	239 (0.16)	241 (0.16)

Hydrodynamic diameter (d_H), polydispersity index (PDI), phosphate-buffered saline (PBS), acetate buffer (Ac.buffer). [a]Formulation performed with n = 3.

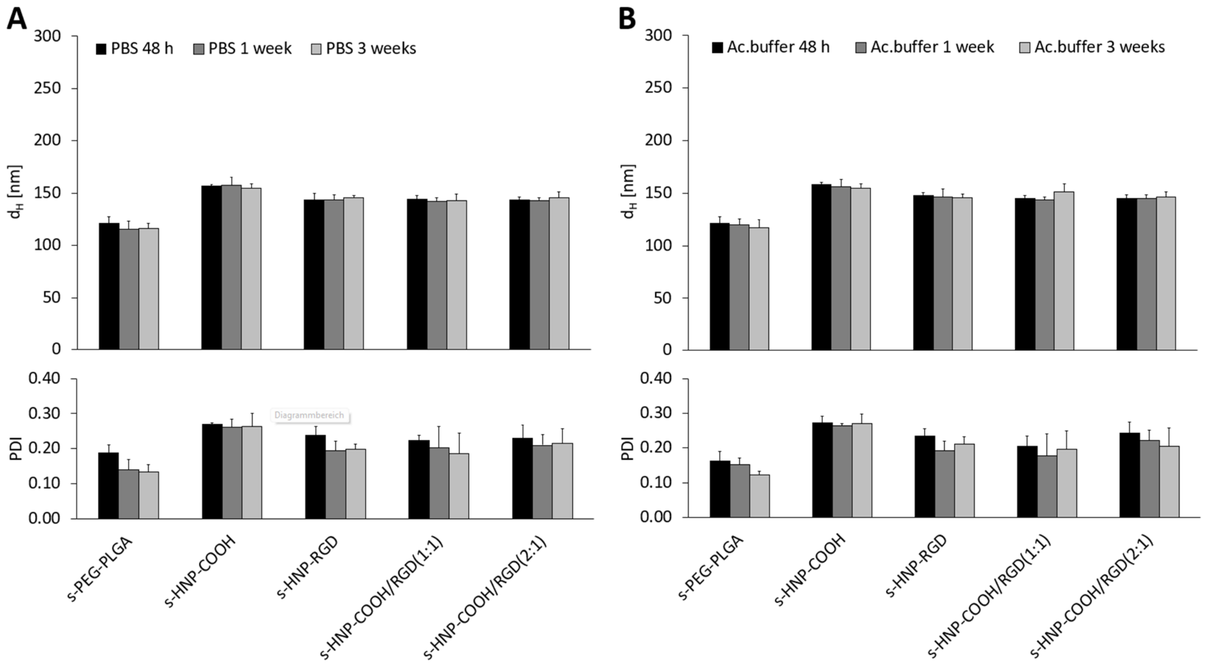

Figure S21: Buffer stability of smaller particles: s-HNPs and s-PEG-PLGA in (**A**) PBS and (**B**) acetate buffer (Ac. buffer).

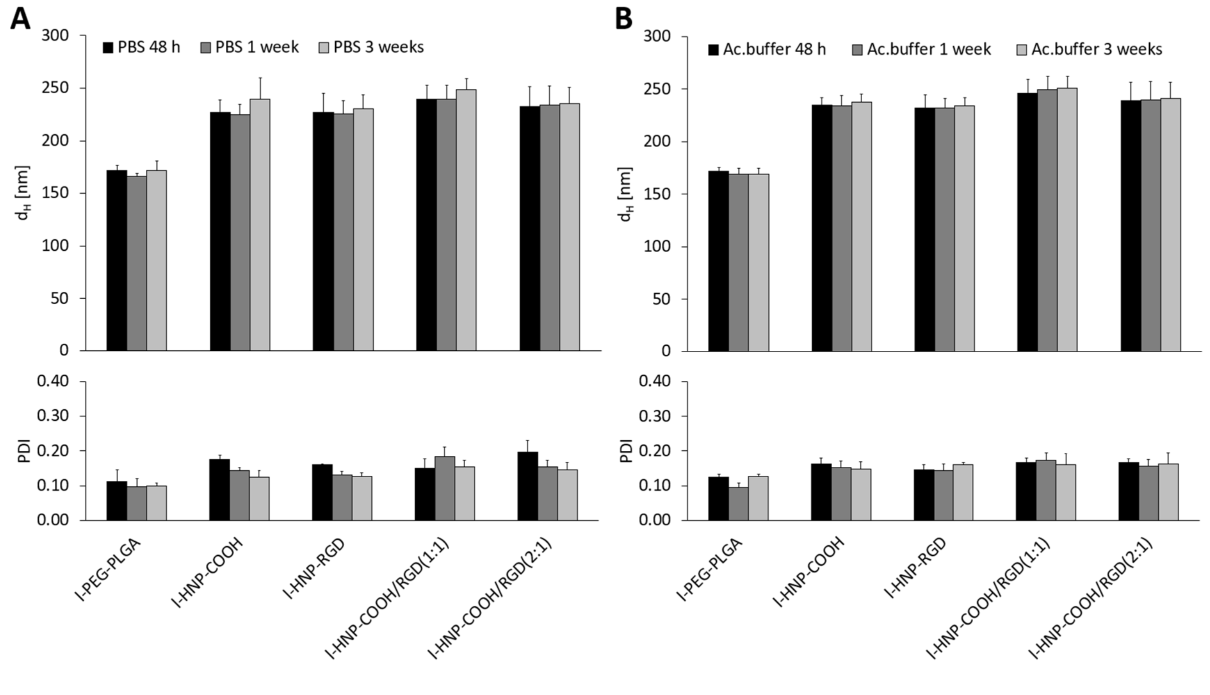

Figure S22: Buffer stability of l-HNPs and l-PEG-PLGA in (**A**) PBS and (**B**) acetate buffer (Ac. buffer).

4.2.7 HPLC analysis of dual loaded HNPS and NPs

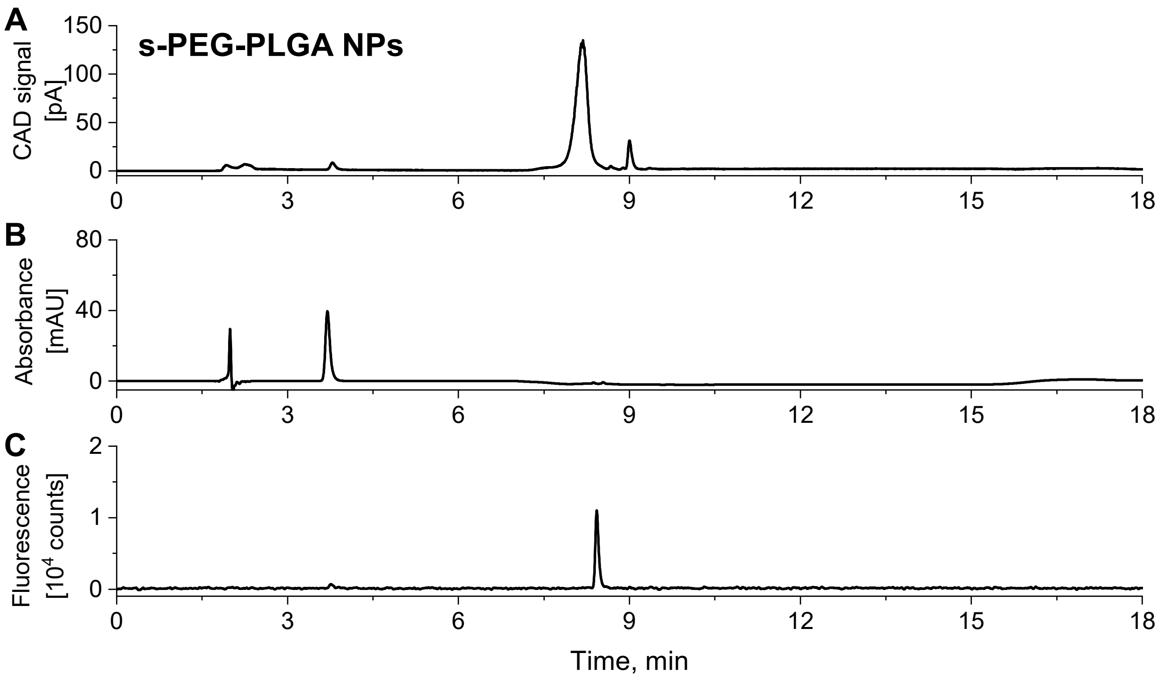

Figure S23: (**A**) Elugram of dual loaded (with the drug BRP-201 and the dye NLO) s-PEG-PLGA NPs recorded by CAD. (**B**) Elugram of s-PEG-PLGA NPs recorded by DAD at 312 nm. The peak at 3.7 min refers to BRP-201. (**C**) Elugram of s-PEG-PLGA NPs recorded by FLD (λ_{ex} = 555 nm, λ_{em} = 592 nm). The peak at 8.4 min refers to NLO. Measurement conditions: Flow rate 0.75 mL min^{-1}, CH_3CN/water with 10 mM ammonium acetate (pH 5.5)/CH_3OH with 10 mM ammonium acetate, gradient conditions can be found in **Figure S2**.

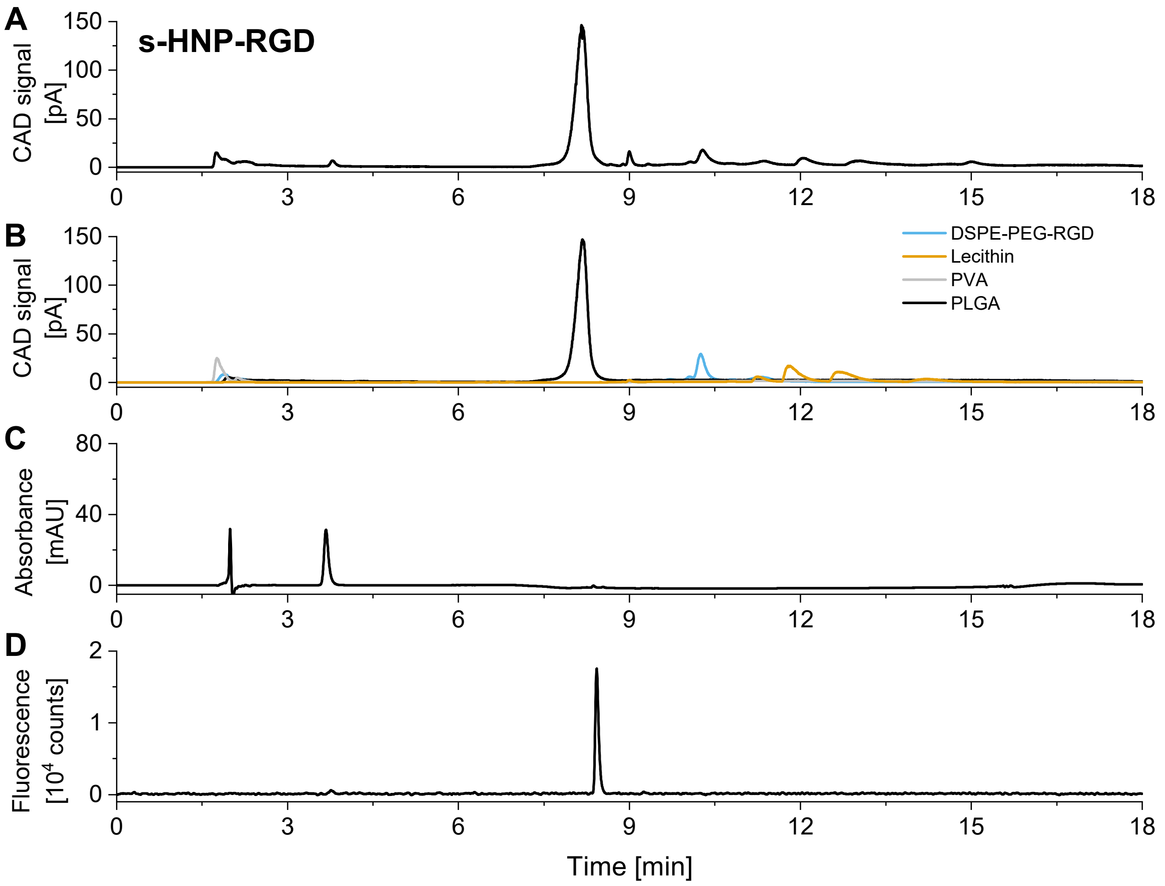

Figure S24: (**A**) Elugram of dual loaded (with the drug BRP-201 and the dye NLO) s-HNP-RGD recorded by CAD. (**B**) Elugrams of PLGA, DSPE-PEG-RGD, lecithin, and PVA standards. For simplicity of interpretation, the signal intensities of DSPE-PEG-RGD, lecithin, and PVA are multiplied with a factor of 0.25. (**C**) Elugram of s-HNP-RGD recorded by DAD at 312 nm. Peak at 3.7 min refers to BRP-201. (**D**) Elugram of s-HNP-RGD recorded by FLD (λ_{ex} = 555 nm, λ_{em} = 592 nm). The peak at 8.4 min refers to NLO. Measurement conditions: Flow rate 0.75 mL min^{-1}, CH_3CN/water with 10 mM ammonium acetate (pH 5.5)/CH_3OH with 10 mM ammonium acetate. The gradient conditions can be found in **Figure S2**.

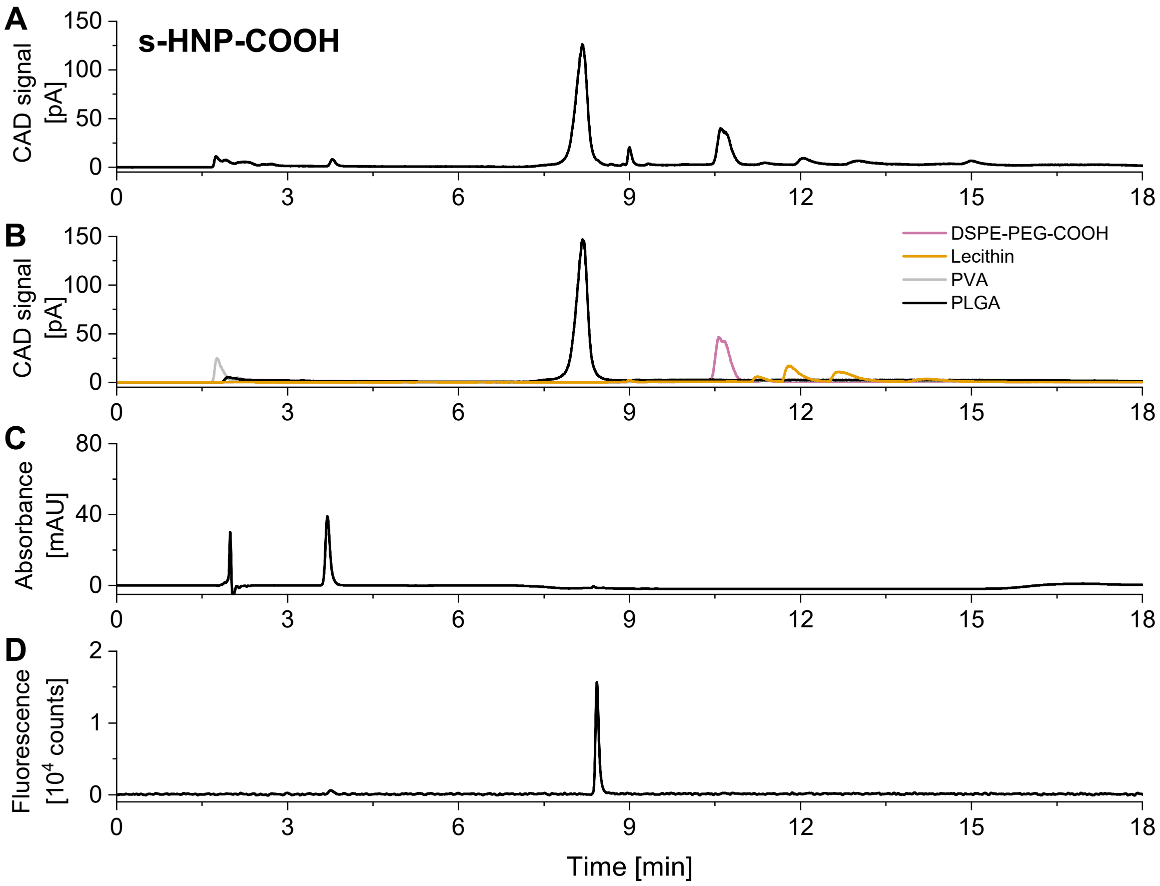

Figure S25: (**A**) Elugram of dual loaded (with the drug BRP-201 and the dye NLO) s-HNP-COOH recorded by CAD. (**B**) Elugrams of PLGA, DSPE-PEG-COOH, lecithin, and PVA standards. For simplicity of interpretation, the signal intensities of DSPE-PEG-COOH, lecithin, and PVA are multiplied with a factor of 0.25. (**C**) Elugram of s-HNP-COOH recorded by DAD at 312 nm. Peak at 3.7 min refers to BRP-201. (**D**) Elugram of s-HNP-COOH recorded by FLD (λ_{ex} = 555 nm, λ_{em} = 592 nm). The peak at 8.4 min refers to NLO. Measurement conditions: Flow rate 0.75 mL min^{-1}, CH_3CN/water with 10 mM ammonium acetate (pH 5.5)/ -CH_3OH with 10 mM ammonium acetate. The gradient conditions can be found in **Figure S2**.

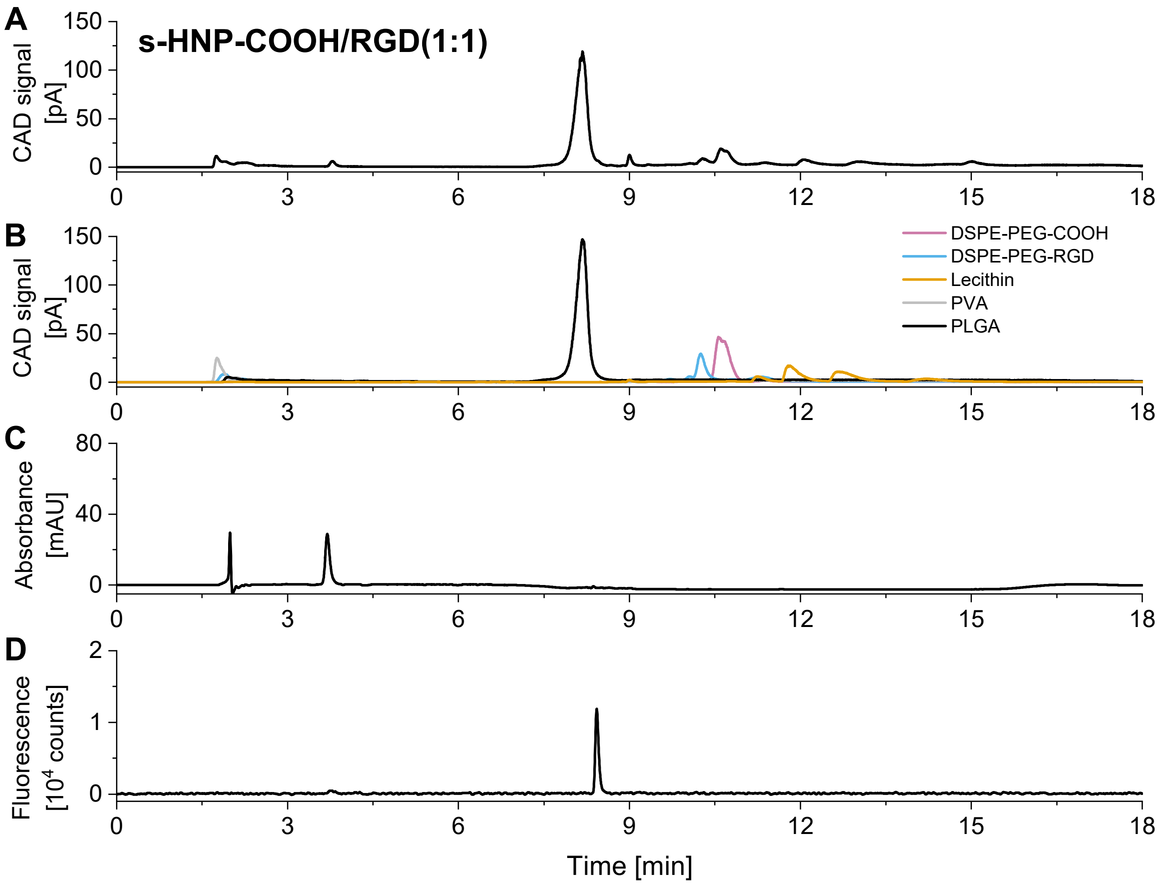

Figure S26: (**A**) Elugram of dual loaded (with the drug BRP-201 and the dye NLO) s-HNP-COOH/RGD(1:1) recorded by CAD. (**B**) Elugrams of PLGA, DSPE-PEG-COOH, DSPE-PEG-RGD, lecithin, and PVA standards. For simplicity of interpretation, the signal intensities of DSPE-PEG-COOH, DSPE-PEG-RGD, lecithin, and PVA are multiplied with a factor of 0.25. (**C**) Elugram of s-HNP-COOH/RGD(1:1) recorded by DAD at 312 nm. Peak at 3.7 min refers to BRP-201. (**D**) Elugram of s-HNP-COOH/RGD(1:1) recorded by FLD (λ_{ex} = 555 nm, λ_{em} = 592 nm). The peak at 8.4 min refers to NLO. Measurement conditions: Flow rate 0.75 mL min^{-1}, CH_3CN/water with 10 mM ammonium acetate (pH 5.5)/ -CH_3OH with 10 mM ammonium acetate. The gradient conditions can be found in **Figure S2**.

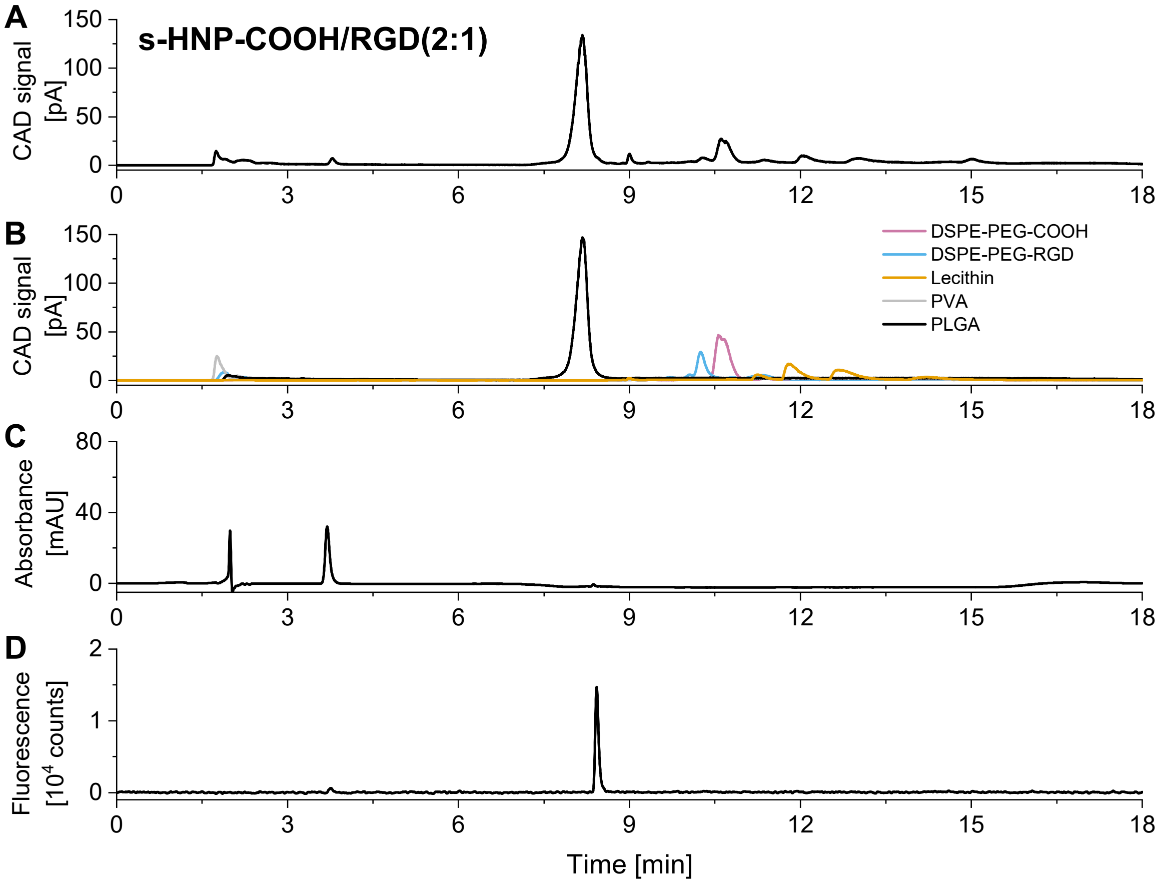

Figure S27: (**A**) Elugram of dual loaded (with the drug BRP-201 and the dye NLO) s-HNP-COOH/RGD(2:1) recorded by CAD. (**B**) Elugrams of PLGA, DSPE-PEG-COOH, DSPE-PEG-RGD, lecithin, and PVA standards. For simplicity of interpretation, the signal intensities of DSPE-PEG-COOH, DSPE-PEG-RGD, lecithin, and PVA are multiplied with a factor of 0.25. (**C**) Elugram of s-HNP-COOH/RGD(2:1) recorded by DAD at 312 nm. Peak at 3.7 min refers to BRP-201. (**D**) Elugram of s-HNP-COOH/RGD(2:1) recorded by FLD (λ_{ex} = 555 nm, λ_{em} = 592 nm). The peak at 8.4 min refers to NLO. Measurement conditions: Flow rate 0.75 mL min^{-1}, CH_3CN/water with 10 mM ammonium acetate (pH 5.5)/CH_3OH with 10 mM ammonium acetate. The gradient conditions can be found in **Figure S2**.

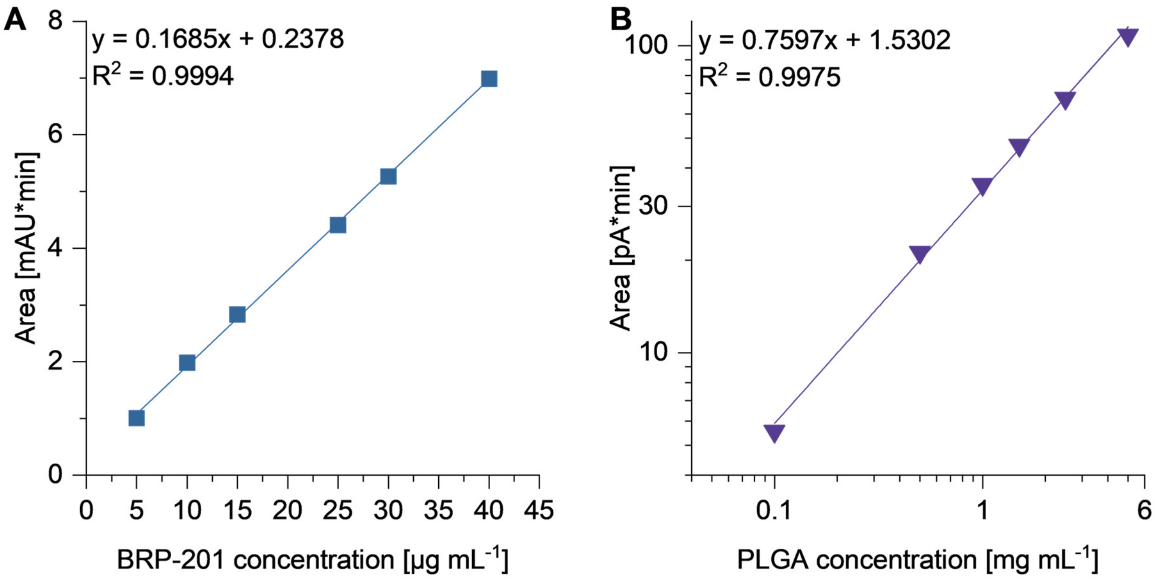

Figure S28: (**A**) Calibration curve for BRP-201 and (**B**) double-logarithmic calibration curve for PLGA presented by plotting peak areas as a function of analyte concentrations. Data were fitted linearly. Data were collected at the same elution conditions as shown in **Figure S2**.

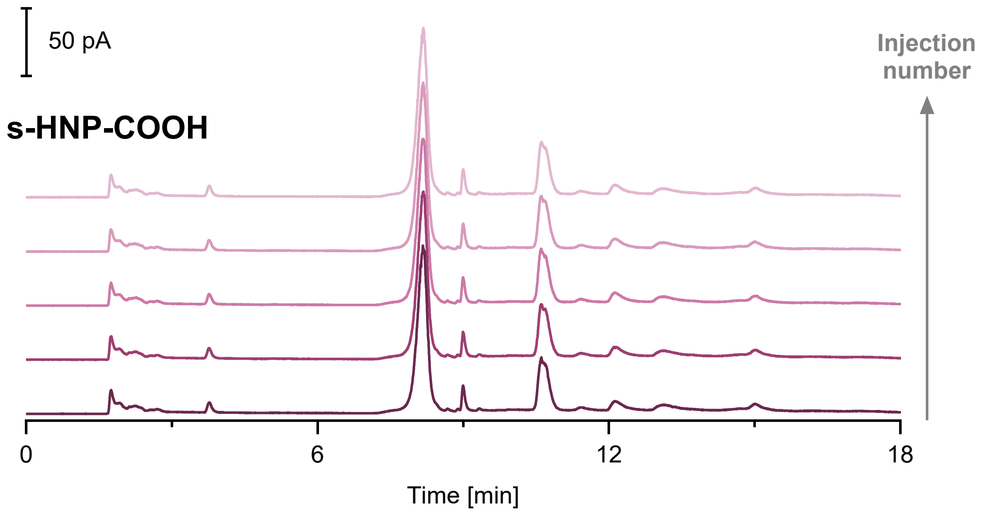

Figure S29: Elution repeatability experiment by five successive injections of s-HNP-COOH recorded by CAD. Measurement conditions: Flow rate 0.75 mL min^{-1}, CH_3CN/water with 10 mM ammonium acetate (pH 5.5)/CH_3OH with 10 mM ammonium acetate. The gradient conditions can be found in **Figure S2**.

Table S9: Compositional analysis of an example batch of formulated s-HNPs and s-NPs. The values were calculated using the calibration data (**Figure S28**).

P#	Sample	m_{lyo} [mg]	BRP-201 [µg mL^{-1}]	PLGA [mg mL^{-1}]	$LC_{BRP-201}$ [%] (rel. to m_{lyo})	$LC_{BRP-201}$ [%] (rel. to $m_{polymer}$)
**Smaller particles**						
P10	s-HNP-COOH	1.114	21.005	0.923	1.89	2.28
P11	s-HNP-RGD	1.322	16.313	1.143	1.23	1.43
P12	s-HNP-COOH/RGD(1:1)	0.949	15.599	0.862	1.64	1.81
P13	s-HNP-COOH/RGD(2:1)	1.156	16.985	1.017	1.47	1.67
P14	s-PEG-PLGA	1.003	21.429	-	2.14	-

Table S10: Repeatability study of the developed method with standard deviation (SD, %) and coefficient of variation (CV, %) for PLGA and DSPE-PEG-COOH retention time and peak area values calculated from five repeated injections of s-HNP-COOH (**Figure S29**).

s-HNP-COOH	PLGA		DSPE-PEG-COOH	
	Retention time [min]	Peak area [pA*min]	Retention time [min]	Peak area [pA*min]
Injection 1	8.179	31.754	10.612	9.373
Injection 2	8.176	31.839	10.611	9.482
Injection 3	8.165	32.042	10.606	9.472
Injection 4	8.180	31.857	10.611	9.535
Injection 5	8.168	31.869	10.607	9.406
**SD, %**	0.60	9.40	0.24	5.74
**CV, %**	0.07	0.29	0.02	0.61

4.2.8 Uptake studies in M1-MDMs

Table S11: Measurement data of the uptake studies of s- and l-HNPs as well as s- and l-PEG-PLGA NPs in M_1-MDMs at 100 µg mL^{-1} with n = 2, reported as mean fluorescence intensity (MFI) corrected for the fluorescence of the particle samples.

P#	Sample	Corrected MFI c_{HNP} = 100 µg mL^{-1}
**Smaller particles**		
P10	s-HNP-COOH	114034
P11	s-HNP-RGD	265855
P12	s-HNP-COOH/RGD(1:1)	267327
P13	s-HNP-COOH/RGD(2:1)	249812
P14	s-PEG-PLGA	254911
**Larger particles**		
P15	l-HNP-COOH	178207
P16	l-HNP-RGD	322568
P17	l-HNP-COOH/RGD(1:1)	320783
P18	l-HNP-COOH/RGD(2:1)	293383
P19	l-PEG-PLGA	326001

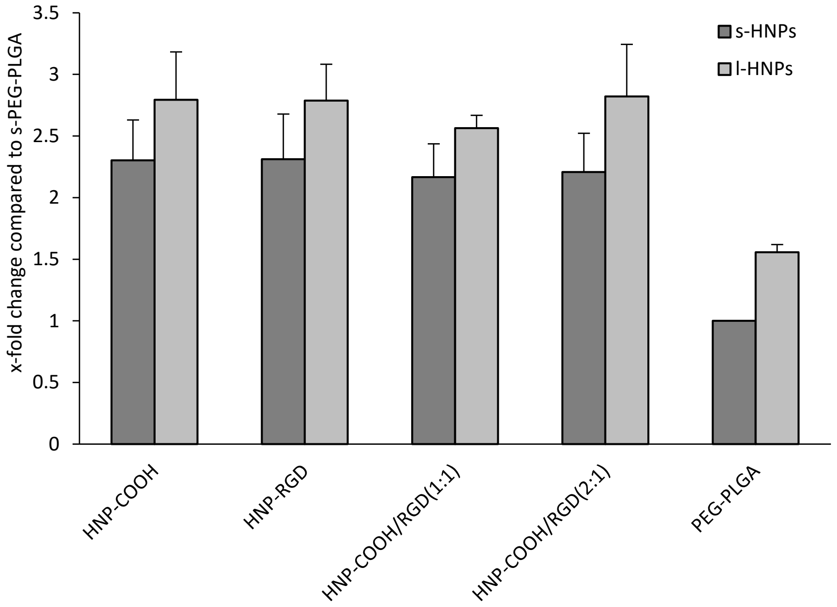

Figure S30: Uptake of the s- and l-HNPs as compared to s- and l-PEG-PLGA NPs (P10-P19) in M_1-MDMs at a concentration of 100 µg mL^{-1} (n = 2), reported as X-fold change as compared to the s-PEG-PLGA NPs.

4.2.9 Investigation of the inhibition efficacy (5-LOX product formation assay)

Table S12: Measurement data of the 5-LOX product formation inhibition of s- and l- HNPs, as well as s- and l-PEG-PLGA NPs.

**P#**	**Sample**	**Inhibition 5-LOX product formation [%] $c_{BRP-201}$ = 0.1 µM**	**Inhibition 5-LOX product formation [%] $c_{BRP-201}$ = 0.3 µM**
**Smaller particles**			
P10	s-HNP-COOH	93,54	48,86
P11	s-HNP-RGD	84,39	32,40
P12	s-HNP-COOH/RGD(1:1)	74,23	66,64
P13	s-HNP-COOH/RGD(2:1)	80,50	44,10
P14	s-PEG-PLGA	95,94	80,39
**Larger particles**			
P15	l-HNP-COOH	/	42,03
P16	l-HNP-RGD	/	40,81
P17	l-HNP-COOH/RGD(1:1)	/	39,28
P18	l-HNP-COOH/RGD(2:1)	/	35,38
P19	l-PEG-PLGA	/	47,01

5 References

[1] K. Zhang, P. M. Jordan, S. Pace, R. K. Hofstetter, M. Werner, X. Chen, O. Werz, *J. Inflamm. Res.* **2022**, 3285-3304.

[2] G. Cinar, J. I. Solomun, P. Mapfumo, A. Traeger, I. Nischang, *Anal. Chim. Acta* **2022**, *1205*, 339741.
